# The hypoxia-inducible factor-1α in stemness and resistance to chemotherapy in gastric cancer: Future directions for therapeutic targeting

**DOI:** 10.3389/fcell.2023.1082057

**Published:** 2023-02-08

**Authors:** Gulnihal Ozcan

**Affiliations:** ^1^ Department of Medical Pharmacology, School of Medicine, Koç University, Istanbul, Turkiye; ^2^ Koç University Research Center for Translational Medicine, Istanbul, Turkiye

**Keywords:** hypoxia-inducible factor-1α, gastric cancer, stemness, chemoresistance, deregulated signaling

## Abstract

Hypoxia-inducible factor-1α (HIF-1α) is a crucial mediator of intra-tumoral heterogeneity, tumor progression, and unresponsiveness to therapy in tumors with hypoxia. Gastric tumors, one of the most aggressive tumors in the clinic, are highly enriched in hypoxic niches, and the degree of hypoxia is strongly correlated with poor survival in gastric cancer patients. Stemness and chemoresistance in gastric cancer are the two root causes of poor patient outcomes. Based on the pivotal role of HIF-1α in stemness and chemoresistance in gastric cancer, the interest in identifying critical molecular targets and strategies for surpassing the action of HIF-1α is expanding. Despite that, the understanding of HIF-1α induced signaling in gastric cancer is far from complete, and the development of efficacious HIF-1α inhibitors bears various challenges. Hence, here we review the molecular mechanisms by which HIF-1α signaling stimulates stemness and chemoresistance in gastric cancer, with the clinical efforts and challenges to translate anti-HIF-1α strategies into the clinic.

## 1 Introduction

Cancer is a dynamic disease that constantly evolves under the influence of genetic instability, epigenetic alterations, and the microenvironment. The evolution of cancer driven by these factors leads to tremendous spatial and temporal heterogeneity within the tumor mass. Tumor heterogeneity plays a crucial role in tumor progression by drawing the road map for stemness and chemoresistance in cancer. Although genetic instability and epigenetic alterations promote tumor heterogeneity, clinical resistance emerges only when the most adaptive clones survive and proliferate despite the selective pressure in the microenvironment. Therefore, the microenvironment has the final word in the construction of the heterogeneity landscape in tumors (Rybinski and Yun, 2016; Senthebane et al., 2017).

The tumor microenvironment is established primarily by the action of biophysical factors. Mainly, insufficient or abnormal vascularity induces gradients of oxygen, pH, nutrients, growth factors, cytokines, interstitial pressure, and blood-borne chemotherapeutics within the tumor mass. The heterogeneous distribution of these factors brings varying characteristics to the tumor cells at different niches in terms of proliferation rate, capabilities for invasion and metastasis, transformation into stem cells or stem-like cells, and propensity to be destructed by immune cells or chemotherapeutics (Trédan et al., 2007; Rybinski and Yun, 2016).

Hypoxia is a critical factor in the tumor microenvironment that shapes the topographic heterogeneity in tumors (Qian and Rankin, 2019). In mammalian tissues, oxygen can travel at a maximum distance of 100–190 µm from capillaries to the cells without being metabolized (Olive et al., 1992). Therefore, maintaining oxygen homeostasis is essential to meet the oxygen requirements for oxidative phosphorylation and to protect cells from oxidative stress. Hence angiogenesis ensures the delivery of oxygen to newly constructed territories and maintains oxygen homeostasis in growing tissues. However, in rapidly proliferating tumors, oxygen homeostasis is disrupted since the rate of angiogenesis cannot keep up with the speed of tumor growth.

Consequent to the imbalance between cancer cell proliferation and angiogenesis, chronic hypoxia or so-called diffusion-limited hypoxia occurs in geometrically distant territories from the capillaries (Kunz and Ibrahim, 2003; Vaupel, 2008; Ruan et al., 2009; Eales et al., 2016; Martin et al., 2016). Moreover, the angiogenic processes may end up with structural and functional abnormalities in tumor microvasculature. The resulting abnormal microcirculation leads to acute hypoxia, which may last from minutes to hours. Then re-oxygenation occurs, and the reactive oxygen species (ROS) generated during the re-oxygenation further potentiate the impact of hypoxia on tumors (Chaplin et al., 1986; Koi and Boland, 2011).

Hypoxia-inducible factor-1α (HIF-1α) is the central mediator in the adaptation of cancer cells to hypoxia in hypoxic niches. HIF-1α reprograms cell metabolism such that the cancer cells shift from oxidative phosphorylation to glycolytic metabolism (Warburg effect), guarantying survival despite a limited source of oxygen in the microenvironment. Furthermore, HIF-1α deregulates intracellular signaling pathways, leading to genomic instability, epithelial-mesenchymal transition (EMT), stemness, and resistance to apoptotic stimuli (Kunz and Ibrahim, 2003; Vaupel, 2008; Ruan et al., 2009; Eales et al., 2016). As a result, tumor cells at hypoxic niches gain survival advantages and play a key role in tumor progression and chemoresistance (Huang et al., 2015; Guo et al., 2016).

Gastric cancer is among the cancers in which hypoxic niches are prevalent (Chen et al., 2014; Miao et al., 2014). HIF-1α has a substantial impact on intracellular cancer signaling pathways in gastric cancer. Upregulation of HIF-1α stimulates angiogenesis, Warburg effect, epithelial-mesenchymal transition, growth factor signaling, replicative immortality, tumor-promoting inflammation, and stemness in gastric cancer. Furthermore, apoptosis in response to anticancer therapy and immune attack is attenuated under the influence of HIF-1α, limiting the efficacy of chemotherapeutics, molecularly targeted agents, and immunotherapy in gastric cancer (Pei et al., 2021; Ucaryilmaz Metin and Ozcan, 2022). Understanding the molecular mechanisms by which HIF-1α induces stemness and chemoresistance is essential to prevent tumor progression and chemoresistance in gastric cancer.

## 2 Gastric cancer and hypoxia

Gastric cancer is among the most malignant tumor types in the clinic. Although it ranks as the fifth most common cancer in the world, it constitutes the fourth most common cause of cancer-related deaths (Sung et al., 2021). Diagnosis, mainly at an advanced stage, and resistance to conventional chemotherapy are the two main reasons for high mortality rates in gastric cancer. Several molecular targeted agents against HER2 and VEGFR, and anti-PD-1 immunotherapeutics, are incorporated into gastric cancer therapy to increase therapeutic success. However, these agents are effective in a small group of advanced/metastatic stage gastric cancer patients with the positivity of target receptors (Joshi and Badgwell, 2021; Lordick et al., 2022). Moreover, gastric tumors also develop resistance to these anticancer agents (Mitani and Kawakami, 2020; Baxter et al., 2021). Therefore, further dissection of the molecular mechanisms that promote chemoresistance in gastric cancer is essential.

Hypoxia and HIF-1α contribute substantially to tumor progression and chemoresistance in gastric cancer. As revealed by nuclear magnetic resonance spectroscopy, gastric tumors are rich in niches with different degrees of hypoxia, from weak to severe. Hypoxia was also detected in the normal gastric mucosa in most gastric cancer patients and displayed a high correlation with decreased overall survival (Bubnovskaya et al., 2014a). Furthermore, gastric tumors with moderate to severe hypoxia are associated with an increased risk of bone metastasis and mortality, even in patients with non-metastatic local tumors (Bubnovskaya et al., 2014b; Bubnovskaya and Osinsky, 2020).

The expression of HIF-1α is highly correlated with malignant phenotype and decreased survival in gastric cancer, like several other cancers (Hui et al., 2002; Branco-Price et al., 2012; Kitajima and Miyazaki, 2013; Kim et al., 2015; Miao et al., 2017). A large-scale meta-analysis in gastric cancer revealed HIF-1α positivity in half of the gastric tumors. Moreover, the HIF-1α expression positively correlated with a higher stage and higher probability of invasion to lymphatic and vascular tissues (Lin et al., 2014). Accordingly, the risk of peritoneal invasion and liver and lymph node metastasis was significantly high, and the 5-year survival rate was significantly low in gastric cancer patients with HIF-1α expressing tumors (Jung et al., 2013; Chen et al., 2014; Miao et al., 2014).

In 90% of gastric cancer cases, the pathological diagnosis is gastric adenocarcinoma (Riquelme et al., 2015). The clinic’s most commonly used histopathological classification system, Lauren, mainly classifies gastric adenocarcinoma into two, intestinal-type and diffuse-type gastric adenocarcinoma (Berlth et al., 2014; Gullo et al., 2018). Differentiation from gastric phenotype to intestinal phenotype (so-called intestinalization) and the presence of glandular structures are characteristics of intestinal-type gastric adenocarcinoma. Approximately 54% of gastric adenocarcinoma patients have intestinal-type cancers. This histopathologic type responds better to chemotherapy than diffuse-type gastric cancer and usually emerges at an older age. Intestinal-type gastric cancer develops from well-defined precancerous lesions with a multi-stage carcinogenesis cascade. This cascade, known as the Correa Cascade, involves chronic inflammation, multifocal atrophic chronic gastritis, intestinal metaplasia, dysplasia, *in situ* carcinoma, and invasive gastric adenocarcinoma sequentially. Risk factors that prompt this process, like *Helicobacter pylori* (*H. pylori*) infection and diet, are relatively well-defined (Correa and Piazuelo, 2012; Cisło et al., 2018).

Diffuse-type gastric adenocarcinoma, on the other hand, is characterized by diffuse infiltration of the stomach wall. Consequently, gland structures become barely or hardly discernible. In contrast to the intestinal-type, non-cohesive undifferentiated mesenchymal cells predominate diffuse-type gastric adenocarcinomas (Krstić and Katić, 2008; Cisło et al., 2018). It emerges at a younger age and has the worst prognosis and poorest response to chemotherapy compared to other gastric cancer types (Riquelme et al., 2015; Ansari et al., 2018). Hereditary diffuse gastric adenocarcinomas, observed in a minority of cases, exhibit autosomal dominant inheritance and are associated with mutations in E-cadherin (CDH1) gene. Although E-cadherin expression is suppressed in non-hereditary diffuse-type gastric adenocarcinoma, the predisposing factors are unknown. Due to the lack of knowledge about precursor lesions, early diagnosis is impossible for most non-hereditary diffuse-type gastric cancers, and therapeutic success is low (Ansari et al., 2018).

The comparative dominance of hypoxic niches in intestinal vs. diffuse-type gastric adenocarcinomas is unknown yet. However, upregulation of HIF-1α was observed more commonly in diffuse-type gastric tumors, compared with intestinal-type gastric tumors (Griffiths et al., 2007; Ma et al., 2007). On the other hand, hypoxia and HIF-1α have prominent roles in the progression of both Lauren types of gastric cancer. HIF-1α exhibited a progressive increase through the successive steps of the Correa cascade of intestinal-type gastric carcinogenesis (Griffiths et al., 2007). In diffuse-type gastric cancer cells, HIF-1α is substantially involved in the EMT, which is crucial for the development and progression of diffuse-type gastric tumors (Matsuoka et al., 2013; Susman et al., 2015; Lee et al., 2017). Therefore, a more exhaustive comprehension of the regulation of HIF-1α in gastric cancer and its role in gastric cancer progression and chemoresistance is required.

## 3 Regulation of HIF-1α

Hypoxia-inducible factors (HIFs) are key transcription factors for adapting normal and cancer cells to hypoxia (Semenza, 2012). HIF isoforms HIF-1, HIF-2, and HIF-3 are all heterodimers of HIF-α and HIF-β subunits. The HIF-1, formed by dimerization of the HIF-1α with HIF-1β, is the leading HIF type in cancer cells. Like other HIF-α isoforms, HIF-1α is sensitive to the oxygen pressure in the microenvironment. In the presence of oxygen, prolyl hydroxylases (PHDs) hydroxylate HIF-1α. After that, ubiquitination occurs and directs HIF-1α to proteasomal degradation. The tumor suppressor protein von Hippel–Lindau (pVHL) takes part in this degradation reaction. HIF-β, on the other hand, is insensitive to oxygen and constitutively expressed in the cell (Kaelin and Ratcliffe, 2008).

Under hypoxia, hydroxylation of HIF-1α by PHDs and its degradation could not occur, leading to upregulation of HIF-1α in the cell. HIF-1α heterodimerizes with HIF-1β in the nucleus. The resulting HIF-1 binds hypoxia-response elements and activates the transcription of several genes required for the adaptation of cells to hypoxia. These HIF-1 stimulated genes are involved in angiogenesis, glycolytic switch, growth, and survival of cancer cells (Muz et al., 2015).

Other than hypoxia, pro-tumorigenic pathways and defective tumor suppressors may lead to the upregulation of HIF-1α. Ras/MAPK and PI3K/Akt/mTOR pathways activated by growth factor receptors can increase HIF-1α expression. Since phosphatase and tensin homolog (PTEN) is a negative regulator of the PI3K/Akt/mTOR pathway, inactivating mutations in PTEN may induce HIF-1α expression *via* upregulation of the PI3K/Akt/mTOR pathway (Muz et al., 2015). Tumor suppressor pVHL is involved in the degradation of HIF-1α, and p53 triggers ubiquitination and proteasomal degradation of HIF-1α in different cancers. Therefore, mutations in pVHL or p53 are associated with increased stability and expression of HIF-1α (Ravi et al., 2000).

## 4 HIF-1α and stemness in cancer

An expanding number of studies that support the cancer stem cell (CSC) model in carcinogenesis, tumor progression, metastasis, and chemoresistance is drawing the attention of anticancer drug discovery studies to CSCs. Hence, characterizing the conditions that induce the development of CSCs and initiation of tumorigenesis by CSCs is under the spotlight of many cancer researchers (Li et al., 2014; Bekaii-Saab and El-Rayes, 2017; Ayob and Ramasamy, 2018; Afify and Seno, 2019). Accordingly, the profound involvement of HIF-1α in stemness presents it as a potential therapeutic target to limit the action of CSCs in cancer.

Hypoxia is a significant factor in the self-renewal and differentiation of stem cells. Studies in embryonic stem cells demonstrated that cells kept under hypoxic conditions maintain stemness and remain undifferentiated, whereas they start to differentiate when switched to a normoxic environment. Several studies noted that low oxygen saturation blocks differentiation and maintains stemness in mesenchymal stem cells (Barnhart and Simon, 2007; Keith and Simon, 2007; Hill et al., 2009). In addition, a hypoxic microenvironment promotes the expansion of hematopoietic stem cells and neuronal stem cells (Morrison et al., 2000; Danet et al., 2003).

Growing evidence reveals that hypoxia also has a prominent role in controlling CSC populations in tumors. CSCs are cancer cells with stem cell-like features such as self-renewal and differentiation into diverse cell populations. CSCs mainly develop from existing stem cells *via* mutations or differentiated cancer cells through dedifferentiation and acquiring stem cell-like properties. Although stemness and cell division are tightly regulated in stem cells, CSCs have the potential for unlimited division and tumorigenesis. Therefore, they are also known as “tumor-initiating cells” (Singh and Settleman, 2010).

Hypoxia has a substantial role in the dedifferentiation of cancer cells to a stem-like phenotype (Carnero and Lleonart, 2016). HIF-1 is a central mediator for the induction and maintenance of stem cell phenotype under hypoxic conditions in several cancers (Tong et al., 2018). HIF-1 activity triggers the expression of important stem cell markers, such as Nanog, Oct4, Sox2, Bmi1, Nestin, LGR5, CD44, CD133, and CD24 in non-stem cancer cells. Thereby, HIF-1 provides stem cell features to regular cancer cells inducing their transformation into CSCs (Heddleston et al., 2009; Matsumoto et al., 2009; Fujikuni et al., 2014; Guo et al., 2016; Liang et al., 2017). Cui et al. reported that hypoxia induces stemness through stabilization of HIF-1ɑ by small ubiquitin-like modifier protease 1 (SENP1) in hepatocellular carcinoma cells, further supporting the substantial effect of hypoxia on cancer cell stemness (Cui et al., 2017).

Hypoxia leads to the selection of the fittest CSC clones with high tumorigenic potential, ensuring the maintenance of tumor stemness (Carnero and Lleonart, 2016). Hypoxic tumor cells isolated from a xenograft model of breast cancer exhibited a predominantly CSC phenotype. When these cells were re-implanted into the mouse, hypoxic tumor cells displayed a more pronounced CSC phenotype than those isolated from the primary xenograft. These cells also exhibited significantly higher tumorigenicity compared with the non-hypoxic cancer cells. The study’s authors identified PI3K/Akt pathway as the requisite for maintaining stemness in these hypoxic tumor cells (Kim et al., 2018). Additionally, hypoxia increases the proliferation of CSCs, as reported in glioblastoma and breast cancer (Heddleston et al., 2009; Lan et al., 2018).

Hypoxia-induced EMT is a crucial mechanism for the maintenance of stemness in CSCs. Hypoxia stimulates the expression of EMT transcription factors Snail, Twist, Zeb1, Slug, and Sip1 in cancer cells *via* transforming growth factor *β* (TGF-β), Notch, and Wnt-β-catenin signaling pathways. These EMT transcription factors, in turn, induce the transcription of stem cell markers, as demonstrated in breast cancer and ovarian cancer models (Seo et al., 2016; Wilson et al., 2020). In addition, ROS and vascular endothelial growth factor (VEGF) upregulated in a hypoxic microenvironment are also involved in the maintenance of stemness (Najafi et al., 2020).

Besides induction and maintenance of stemness of cancer cells, high expression of stem cell markers is associated with a higher propensity of CSCs to migrate toward hypoxic areas (Barnhart and Simon, 2007; Keith and Simon, 2007; Hill et al., 2009). Moreover, HIF-1 leads to the recruitment of stem cells to hypoxic areas by inducing the release of chemokine 12 (CXCL12) (formerly known as stromal-derived factor-1ɑ) into the tumor microenvironment (Das et al., 2008). Due to the hypoxia-induced stemness, proliferation, and recruitment of CSCs, hypoxic niches are rich in CSCs, which introduces these niches as potent drivers of tumorigenesis, invasion, and metastasis (Carnero and Lleonart, 2016).

Equipment of CSCs with several chemoresistance mechanisms in hypoxic environments further extends the handicaps that hypoxia poses in cancer treatment. Under hypoxia, HIF-1α induced Warburg effect decreases the intracellular ROS concentration that would be higher in the case of oxidative phosphorylation. Thus, the cytotoxic effects of DNA damaging chemotherapeutics and radiotherapy decline. Additionally, the glycolytic switch increases the intracellular concentration of glutathione (GSH) (Najafi et al., 2020), which suppresses the action of several chemotherapeutics, such as platinum derivatives and anthracyclines (Traverso et al., 2013). Moreover, hypoxia increases the expression of drug efflux pumps, mainly ABCB1 (or P-gp), and the telomerase activity, leading to multidrug resistance in CSCs. Primarily HIF-1α mediates these effects. Hypoxia-induced plasticity allows CSCs to switch between EMT and mesenchymal-epithelial transition (MET), bringing a survival advantage to CSCs in dynamically changing environments. (Najafi et al., 2020). Furthermore, chronic hypoxia induces senescence in CSCs, building additional barriers to the action of anticancer therapies which act on highly proliferating cells (Carcereri de Prati et al., 2017; Emami Nejad et al., 2021).

## 5 HIF-1α and stemness in gastric cancer

Stemness in gastric cancer is a significant inducer of tumor progression and chemoresistance. Gastric CSCs can differentiate into multiple cell types at distinct regions of the stomach. These cells can evade immunity and constitute a tumor microenvironment that suppresses immunosurveillance. This tumor microenvironment is also fertile soil for the maintenance of stemness and transformation of more gastric cancer cells into gastric CSCs. Furthermore, gastric CSCs are involved in metastasis and recurrence in gastric cancer (Becerril-Rico et al., 2021; Yang et al., 2022). Therefore, targeting stemness is rising as a new therapeutic strategy in gastric cancer (Rao et al., 2022).

HIF-1α is a key stimulator of stemness in gastric cancer. Hypoxia and increased HIF-1α are associated with increased expression of stem cell markers in gastric cancer cell models and patient samples. Guo et al. exposed BGC823 and SGC7901 gastric cancer cells to hypoxic conditions with 1% oxygen. They observed that the expression of EMT markers and critical stem cell markers Oct4, Sox2, and Bmi1 increased significantly compared to the cells kept under normoxic conditions. They validated the increased expression of the stem cell markers at both the gene and protein levels. An increase in proliferation, invasion, migration, and clonogenicity accompanied the increase in the EMT and stem cell markers. These findings pointed out the role of hypoxia in the induction of stemness and a more aggressive phenotype in gastric cancer cells (Guo et al., 2016).

Miao et al. investigated the gastrectomy specimens from 175 gastric cancer patients and reported that HIF-1α expression was associated with increased expression of stem cell markers Oct4 and Nestin in both differentiated and undifferentiated gastric tumors. The survival rates were significantly lower in patients with a high expression of HIF-1α, Oct4, and Nestin. When the authors exposed primary gastric cancer cell models they developed from gastrectomy specimens to hypoxia, they observed 8.7- and 5.1-fold increase in stem/progenitor cell-specific markers LGR5 and CD44, respectively, an increased self-renewal capability and a decrease in the differentiation of gastric stem cells. Knocking down HIF-1α reverted these changes, showing the involvement of HIF-1α in the proliferation of gastric cancer stem cells and maintenance of stemness (Miao et al., 2014).

Furthermore, peritoneal dissemination was significantly high in patients whose tumors displayed an increased expression of HIF-1α but not HIF-2α. Peritoneal dissemination of gastric cancers is thought to occur by the entry of gastric CSCs through peritoneal milky spots, which are small lymphoid tissues in the peritoneum. Since peritoneal milky spots are considered hypoxic tissues, Miao et al. investigated whether peritoneal milky spots operate as hypoxic niches facilitating the homing of gastric CSCs to the peritoneum. In a mice peritoneal dissemination model, they observed that peritoneal milky spots were rich in gastric CSCs. When they injected gastric CSCs with depleted HIF-1α or replete HIF-1α into the peritoneum of mice, the rate of peritoneal dissemination was significantly low in the HIF-1α depleted group (Miao et al., 2014). All these findings pointed out the significance of HIF-1α in stem cell-driven metastasis in gastric cancer.

Cell adhesion molecule CD44 is a key regulator of stemness, metastasis, and resistance to chemoradiotherapy in several tumors (Yan et al., 2015). Furthermore, expression of CD44 is associated with self-renewal and generation of differentiated progeny in gastric cancer cells. Therefore, it is regarded as a cell surface marker to identify gastric CSCs (Takaishi et al., 2009). Under hypoxic conditions, CD44 expression increased by 3-fold and 2.2-fold, respectively, in SGC7901 and BGC823 gastric cancer cells. In addition, these cells’ survival and invasion capabilities increased under hypoxia. Since rapamycin, which down-regulates HIF-1α, could revert these changes, HIF-1α was suggested as the mediator of CD44 induction and stemness in gastric cancer cells (Liang et al., 2017). However, the study did not investigate the effect of explicitly suppressing HIF-1α. Therefore, the direct link between HIF-1α and CD44 induction needs further validation.

CD24 is a putative CSC marker which is upregulated in several tumors and is associated with an aggressive phenotype. The positivity of CD24 is associated with tumor progression, invasion, lymphatic metastasis, and, consequently, a dismal prognosis in both Lauren types of gastric cancer. Although CD24 expression displayed a heterogenous pattern in ascites samples from gastric cancer patients, studies in TMK-1 (poorly differentiated), 44As3 (signet ring cell/diffuse-type), and NCI-N87 (intestinal-type) gastric cancer cell lines showed that hypoxic conditions could induce the expression of CD24 in CD24 negative cells leading up to a 60-fold increase in CD24. These findings suggested that the heterogeneity in the CD24 expression may result from a heterogenous pattern of hypoxia within the tumor mass or ascitic environment. Moreover, CD24 expression increased the migration rate of TMK-1 cells, and the knockdown of CD24 suppressed the hypoxia-induced invasion in TMK-1 cells. The authors suggested both HIF-1α and HIF-2α as mediators of hypoxia-induced CD24 expression in TMK-1 cells (Fujikuni et al., 2014).

CD133 is another cell surface protein used as a CSC marker in several cancers. Hypoxia regulates CD133 expression both in healthy and cancer tissues. Contrary to the positive regulatory role of HIF-1α on CD44 and CD24, HIF-1α exhibited a negative regulatory effect on CD133 in a panel of gastric cancer cell lines. HIF-1α expression was also negatively correlated with CD133 expression in gastric tumor specimens. These observations contradict the positive regulatory role of HIF-1α on CD133 in other tumors like glioma suggesting a context-dependent regulation of CD133 by HIF-1α (Matsumoto et al., 2009).

A later study suggested a relationship between the pattern of CD133 expression in immunohistology staining and HIF-1α positivity. In differentiated gastric tumors, CD133 was predominantly expressed at the luminal site of the cancer cells and correlated with a lower rate of HIF-1α positivity. However, in undifferentiated gastric tumors, CD133 expression was predominant at the cytoplasm and associated with a higher rate of HIF-1α positivity. Furthermore, cytoplasmic expression of CD133 was associated with a higher rate of peritoneal, lymphatic, and hematogenous metastasis, poor prognosis, and chemoresistance compared with the gastric tumors that express CD133 luminally or that are CD133 negative. Moreover, gastric cancer patients with both HIF-1α- and CD133-positive tumors displayed the poorest prognosis (Hashimoto et al., 2014). Nevertheless, further studies are needed to uncover the mechanisms for this context-dependent and subcellular compartment-dependent regulation of CD133, the influence that CD133 brings upon gastric cancer cells, and the regulation of CD133 by HIF-1α.

**FIGURE 1:**
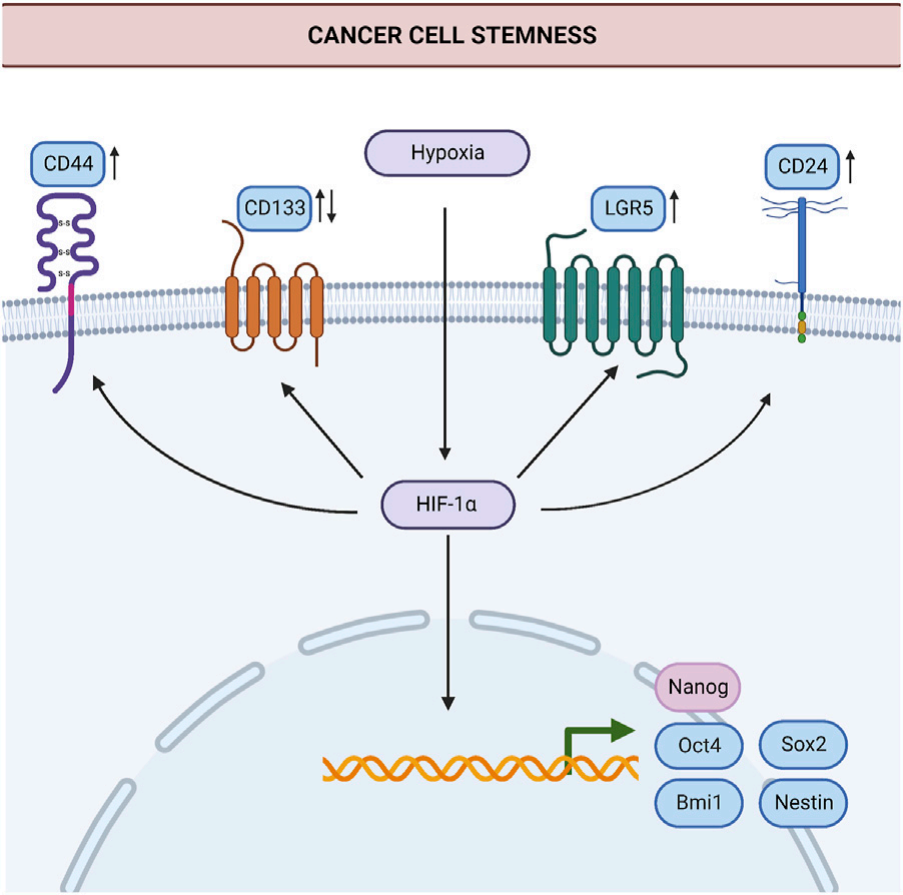
Induction of stemness by hypoxia in gastric cancer. HIF-1α induces the expression of stem cell markers such as Oct4, Sox2, Bmi1, Nestin, Nanog, LGR5, CD44, and CD24 in cancer cells. Thereby, HIF-1α contributes to the generation of new cancer stem cells and the maintenance of stemness. HIF-1α may suppress or increase the expression of CD133 in gastric cancer, in a context-dependent manner. Processes for which there is specific evidence in gastric cancer are shown in blue. Processes common in different cancers are shown in light pink. Upregulation or downregulation of specific proteins are shown with an upward or downward arrow, respectively. Abbreviations: Bmi1, B-cell-specific Moloney murine leukemia virus integration site-1; HIF-1α, Hypoxia-inducible factor 1α; LGR5, Leucine Rich Repeat Containing G Protein-Coupled Receptor five; Oct-4, Octamer-binding transcription factor 4; Sox2, SRY (sex determining region Y)-box 2. *Created with BioRender.com.*

In summary, all the studies mentioned here put forth HIF-1α as a potential target to surpass CSC-driven tumor progression, metastasis, and chemoresistance. The key stem-cell markers affected by HIF-1α in the studies mentioned above are summarized in Figure 1 to represent the current knowledge on the link between HIF-1α and stemness in gastric cancer.

## 6 Hypoxia and chemoresistance in cancer

The understanding that cancer is a systemic but not a localized disease raised the efforts to develop systemic anticancer therapies, leading to the development of the first cancer chemotherapeutics starting from 1940’s (DeVita and Chu, 2008). Despite now we have a plethora of systemic chemotherapeutics potent and efficacious in the first-line treatment of cancer, chemoresistance is still a significant handicap in cancer therapy. Progress in identifying underlying molecular mechanisms and efforts to block critical targets paved the way for developing molecular-targeted agents targeting deregulated signaling pathways in cancer. Incorporating these agents into the chemotherapy regimens over the years increased the response rates in patients resistant to conventional chemotherapeutics. However, acquired resistance to these agents is also a challenge to surpass (Vasan et al., 2019).

The dynamic nature of cancer signaling due to genetic and epigenetic alterations makes it a tough battle to overcome chemoresistance (Nikolaou et al., 2018). The hypoxic microenvironment adds other front lines to this battle by preventing the drug action, from the penetration of anticancer drugs into tumor tissue to the induction of cell death (Jing et al., 2019). Hence, tumor hypoxia is a crucial determinant for responsiveness to neoadjuvant and adjuvant chemotherapy in cancer (Bubnovskaya and Osinsky, 2020).

In rapidly proliferating tumors, penetration distance from capillaries to the tumor cells increases, impairing the penetration of chemotherapeutics to distant territories where hypoxia is common (Trédan et al., 2007). Additionally, glycolytic shift and increased lactate production in hypoxic niches create an acidic microenvironment that disrupts the cellular uptake of anticancer drugs. For instance, weak basic chemotherapeutics such as doxorubicin, vincristine, and mitoxantrone become ionized and trapped in the acidic extracellular environment, leading to decreased uptake of these agents into the tumor cells (Tannock and Rotin, 1989; Mahoney et al., 2003; Gerweck et al., 2006). Drug efflux also increases under hypoxia due to the HIF-1α-induced expression of drug efflux pumps (Jing et al., 2019). Hypoxia-induced cell cycle arrest and low proliferation rate at hypoxic niches further strengthen the impact of hypoxia in chemoresistance since conventional chemotherapeutics primarily act on rapidly proliferating cells (Amellem and Pettersen, 1991; Raz et al., 2014; Valencia-Cervantes et al., 2019). Moreover, resistance to apoptosis, genetic instability, EMT, and CSCs induced by hypoxia reinforce the development of chemoresistance in tumors (Ruan et al., 2009).

## 7 HIF-1α and chemoresistance in gastric cancer

**FIGURE 2:**
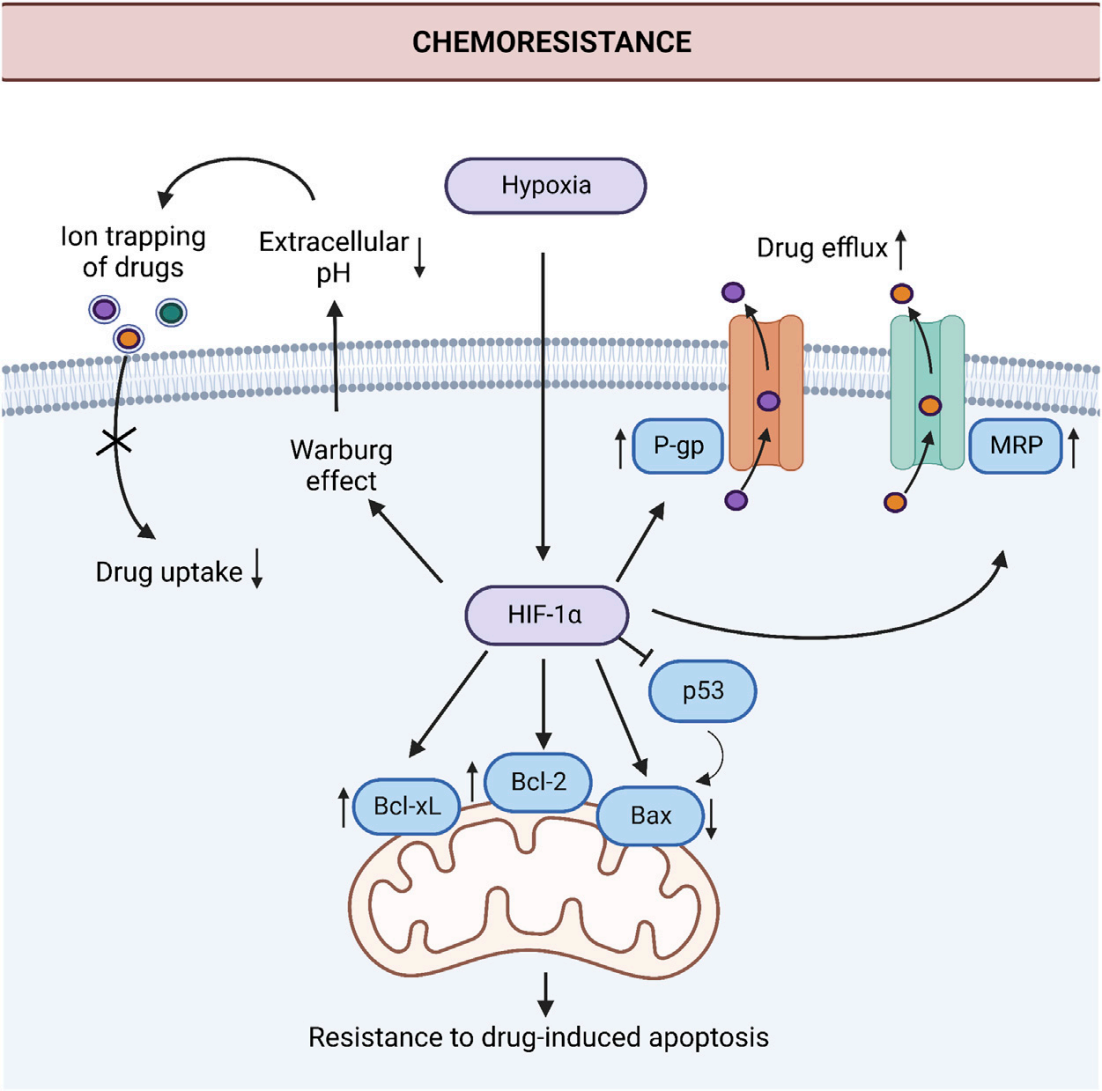
Induction of chemoresistance by hypoxia in gastric cancer. HIF-1α induces chemoresistance *via* the downregulation of proapoptotic protein Bax, upregulation of anti-apoptotic proteins Bcl-2 and Bcl-xL, and drug efflux pumps P-gp and MRP in gastric cancer cells. Additionally, the Warburg effect increases extracellular acidity under hypoxic conditions. This acidic environment leads to ion trapping of the drugs in the extracellular space and decreases drug uptake. Abbreviations: Bax, Bcl-2-associated X protein; Bcl-2, anti-apoptotic protein B-cell lymphoma 2; Bcl-xL, B-cell lymphoma extra-large; HIF-1α, Hypoxia-inducible factor 1α; MRP, The human multidrug resistance-associated protein; P-gp, P-glycoprotein; p53, Tumor protein p53. *Created with BioRender.com.*

Gastric cancer is one of the most aggressive cancers at which the extent of the challenge posed by chemoresistance is massive (Marin et al., 2020). Despite that, knowledge of the mechanisms that operate chemoresistance in gastric cancer is limited. Consistent with the relative sparsity of studies in gastric cancer compared with other immensely studied cancers, the number of studies that dissect the role of hypoxia and HIF-1α in chemoresistance is relatively low in gastric cancer. Studies investigating the role of increased drug efflux and resistance to apoptosis under hypoxia dominate the current literature on hypoxia-induced chemoresistance in gastric cancer. Figure 2 summarizes these mechanisms.

Liu et al. (2008) investigated the inducer role of HIF-1α in chemoresistance to doxorubicin and vincristine, two widely used conventional chemotherapeutics, in gastric cancer *in vitro* models. When they incubated the SGC7901 gastric cancer cells in a hypoxic microenvironment or overexpressed HIF-1α, the efflux of doxorubicin increased significantly, decreasing the intracellular accumulation of the drug. The authors assigned these observations to the upregulation of two critical efflux pumps, P-gp and MRP1, with exposure to hypoxia or exogenous expression of HIF-1α. Additionally, hypoxia led to the upregulation of anti-apoptotic protein Bcl-2 and downregulation of proapoptotic protein Bax, suppressing the apoptotic effect of chemotherapeutics. As a result, the IC50 of doxorubicin and vincristine increased by 1.76- and 8.9-fold, respectively. Furthermore, the IC50 of these chemotherapeutics increased more dramatically in HIF-1α overexpressing SGC7901 cells, with a 2.36-fold increase for doxorubicin and a 14.8-fold increase for vincristine. The authors validated the role of HIF-1α since silencing the HIF-1α with siRNAs reverted chemoresistance in SGC7901 cells. With further studies, the research group established subcutaneous mouse models with vincristine-resistant SGC7901 cells. Injection of HIF-1α targeting siRNAs decreased the tumor volume and increased sensitivity to chemotherapy in these *in vivo* models. Furthermore, HIF-1α targeting siRNAs reduced the expression of P-gp, MRP, and Bcl-2 and increased the expression of Bax in tumors from mouse models (Liu et al., 2008). Hence, *in vivo* findings validated the *in vitro* findings in gastric cancer cells, strengthening the significance of HIF-1α in gastric cancer.

Okazaki et al. followed a different approach and examined the changes in the expression of HIF-1α and other hypoxia-associated genes in chemoresistant gastric cancer cells established by exposing MKN45 cells to paclitaxel (MKN45-PTX). Not only HIF-1α but also HIF-1α-regulated proteins VEGF and glycolytic enzyme pyruvate kinase M1 (PKM1) were upregulated in these cells. The authors also observed the upregulation of P-gp, MRP, and anti-apoptotic protein Bcl-xL in paclitaxel-resistant gastric cancer cells. Conversely, Bax and Caspase-3 expression were lower in MKN45-PTX cells compared with parental MKN45 cells. Xenograft models established with MKN45 or paclitaxel-resistant MKN45 (MKN45-PTX) exhibited similar findings with *in vitro* studies. For instance, the expression of VEGF and PKM1 was higher in tumors from MKN45-PTX *in vivo* model. These findings suggest that exposure to chemotherapeutics or a chemoresistant phenotype may correlate with an increased expression of HIF-1α and HIF-1α regulated genes in gastric cancer. However, although the increase in hypoxia genes was parallel to the rise in genes associated with chemoresistance, the study design does not allow the establishment of mechanistic links between HIF-1α and chemoresistance in the study. Therefore, whether HIF-1α acts as a mediator of the upregulation in P-gp, MRP, or anti-apoptotic proteins in MKN45 gastric cancer cells exposed to paclitaxel needs validation with knockdown/out experiments (Okazaki et al., 2018).

Rohwer et al. (2010) took advantage of knockdown strategies for a robust investigation of the role of HIF-1α in chemoresistance in gastric cancer. When they knocked down the expression of HIF-1α in AGS gastric cancer cell lines, they observed increased sensitivity to 5-fluorouracil (5-FU), a commonly used chemotherapeutic in gastrointestinal cancers, and cisplatin, a widely used potent chemotherapeutic in several cancers. On the contrary, overexpression of HIF-1α induced resistance to chemotherapeutics in these cells. In HIF-1α deficient AGS cells, the efficacy of 5-FU to induce apoptosis through p53 and cell-cycle arrest through p21 increased substantially. Concomitant silencing of p53 with HIF-1α reduced the chemo-sensitization achieved by silencing HIF-1α *per se* in AGS cells. Moreover, silencing of HIF-1α in a p53-mutant gastric cancer cell line MKN28 could not achieve chemo-sensitization to 5-FU, nor the silencing of p53 alone. However, concomitant restoration of functional p53 with the silencing of HIF-1α allowed chemo-sensitization of these cells to 5-FU. These findings suggested that HIF-1α blocks cell-cycle arrest and apoptosis induced by p53 after exposure to 5-FU, and an intact p53 is required to revert chemoresistance by suppressing HIF-1α action in gastric cancer cells. Further experiments suggested that HIF-1α may decrease the p53 activity through suppression of ROS production, which is an efficient activator of the p53 function (Rohwer et al., 2010). Supporting the role of HIF-1α in chemoresistance to 5-FU in gastric cancer, Nakamura et al. reported that the survival rate under 5-FU treatment was significantly lower in advanced-stage gastric cancer patients with diffuse expression of HIF-1α in the tumor, compared to the patients with HIF-1α negative tumors or HIF-1α positivity only in the invasive margins of the tumors (Nakamura et al., 2009).

A more recent study by Zhang et al. (2014) not only strengthened the evidence that the HIF-1α upregulates drug efflux pumps and downregulates apoptotic proteins but also proposed Krüppel-like factor 8 (KLF8) as a mediator for these responses in gastric cancer cells. KLF8 is a transcription factor associated with oncogenic transformation in several cancers. Since the lack of VHL, which results in the stabilization of HIF-1α, is related to the overexpression of KLF8 in renal cell carcinoma, the research group investigated whether KLF8 is involved in hypoxia-induced chemoresistance in gastric cancer. They observed that the expression of KLF8 increased substantially under hypoxic conditions in parallel to HIF-1α in MKN45, MKN28, and SGC7901 gastric cancer cells. Conversely, silencing of HIF-1α downregulated KLF8 in these cells. Moreover, ectopic expression of KLF8 induced chemoresistance to cisplatin, 5-FU, vincristine, and doxorubicin in SGC7901 cells. Further investigations revealed that KLF8 increases the expression of P-gp and Bcl-2 and decreases the expression of Bax and Caspase-3 in gastric cancer cells. Hence, they demonstrated that KLF8 is a mediator of hypoxia-induced chemoresistance by increasing drug efflux from cancer cells and suppressing apoptosis in response to chemotherapeutics (Zhang et al., 2014).

## 8 Therapeutic targeting of HIF-1α

**FIGURE 3:**
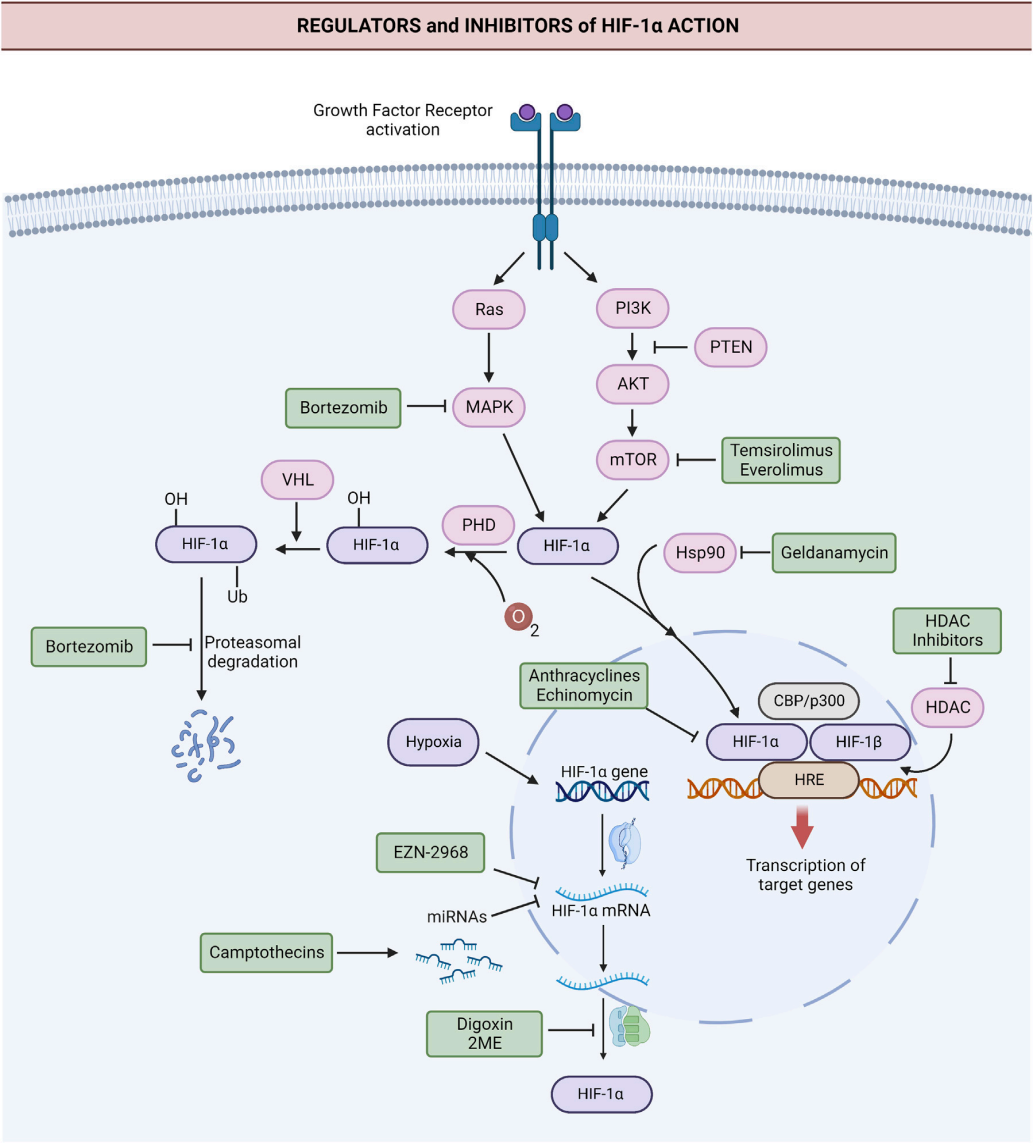
Targets of the major anti-HIF-1α agents tested in clinical trials. Anti-HIF-1α agents directly or indirectly inhibit HIF-1α *via* different mechanisms. Anti-HIF-1α oligonucleotide EZN-2968 leads to the degradation of HIF-1α mRNA. Camptothecins irinotecan and topotecan downregulate HIF-1α *via* HIF-1α-targeting miRNAs. Digoxin and 2-methoxyestradiol (2ME2) suppress the translation of HIF-1α. Rapamycin analogs, like everolimus and temsirolimus, downregulate HIF-1α by inhibiting mTOR. Hsp90 inhibitor geldanamycin and HDAC inhibitors destabilize HIF-1α. Anthracyclines and the peptide antibiotic echinomycin block the binding of HIF-1α to DNA and thus inhibit HIF-1α action. Proteosome inhibitor bortezomib blocks the degradation of ubiquitinated HIF-1α and leads to the accumulation of HIF-1α in an inactive form. Additionally, it suppresses the expression of HIF-1α *via* inhibition of PI3K/Akt/mTOR and MAPK pathways. Abbreviations: Akt, Protein kinase B; CBP, cyclic AMP response element-binding protein; GF, Growth factor; HDAC, Histone deacetylase; HIF-1α, Hypoxia-inducible factor 1α; HIF-1β, Hypoxia-inducible factor 1β; HRE, Hypoxia response elements; HSP90, Heat shock protein 90; MAPK, Mitogen-activated protein kinase; mRNA, Messenger RNA; mTOR, The mammalian target of rapamycin; p300, transcriptional coactivator p300; PHD, prolyl hydroxylase; PI3K, Phosphoinositide 3-kinase; Ras, Ras GTPase; Ub, ubiquitin tag. *Created with BioRender.com.*

The substantial impact of HIF-1α on hallmarks of cancer, stemness, and chemoresistance makes it an attractive target in cancer therapy. Many drugs with indirect inhibitory action on HIF-1α have been tested in clinical trials for the past 20 years, and efforts to develop direct HIF-1α inhibitors continue (Burroughs et al., 2013; Masoud and Li, 2015; Özcan and Keskin, 2020). The major ones registered at ClinicalTrials.gov (US National Library of Medicine, 2007) are listed in Table 1, and their mechanisms of HIF-1α inhibition are shown in Figure 3.

**TABLE 1:** Selected clinical trials registered to ClinicalTrials.gov testing HIF-1α inhibitors in cancer.

Start-end date	Drugs	Condition	Phase	Status	Trial id
EZN-2968 (Anti-sense HIF-1α oligonucleotide)					
2007–2011	EZN-2968	Advanced solid tumors or lymphoma	I	Compl.	NCT00466583
2010–2013	EZN-2968	Neoplasms, liver metastasis	I	Compl.	NCT01120288
2016–2018	EZN-2968	Hepatocellular carcinoma	I	Compl.	NCT02564614
Camptothecins (downregulate HIF-1α *via* HIF-1α-targeting miRNAs)					
2005–2010	Oral Topotecan	Refractory advanced solid neoplasms expressing HIF-1a	I	Compl.	NCT00117013
2007–2012	Topotecan in combination with Cisplatin and Bevacizumab	Recurrent/persistent cervical cancer	II	Compl.	NCT00548418
Rapamycin analogs (suppress the expression of HIF-1α *via* inhibition of mTOR)					
2009–2012	RAD001 in combination with Sorafenib	Advanced solid tumors	I/II	Suspend.	NCT01226056
2010–2015	RAD001 (Everolimus) in combination with FOLFOX and Bevacizumab	Colorectal carcinoma	I/II	Compl.	NCT01047293
2011–2013	Irinotecan and Rapamycin	Refractory solid tumors in children	I	Compl.	NCT01282697
Digoxin (suppress the translation of HIF-1α)					
2010–2013	Digoxin	Prostate cancer	II	Compl.	NCT01162135
2013–2016	Digoxin	Breast cancer	II	Compl.	NCT01763931
2019-	Digoxin in combination with Metformin and Simvastatin	Advanced solid tumors	I	Recruit.	NCT03889795
2021-	Digoxin in combination with FOLFIRINOX	Resectable pancreatic cancer	II	Recruit.	NCT04141995
2-Methoxyestradiol (suppress the translation of HIF-1α)					
2006–2008	2-Methoxyestradiol Nanocrystal Colloidal Dispersion (Panzem)	Prostate cancer	II	Compl.	NCT00394810
HDAC inhibitor Romidepsin (destabilize HIF-1α)					
2012–2018	Romidepsin	Lymphoma, chronic lymphocytic leukemia, or solid tumors with liver dysfunction	I	Active, not recruit.	NCT01638533
2013–2022	Romidepsin and Pralatrexate	Lymphoid malignancies	I/II	Compl.	NCT01947140
2016–2021	Romidepsin in combination with oral Azacytidine (CC-486) and Pembrolizumab (MK-3475)	Advanced colorectal cancer	I	Compl.	NCT02512172

Compl.: completed, Recruit.: recruiting, Suspend.: suspended.

An anti-HIF-1α oligonucleotide EZN-2968 had been tested in advanced solid tumors, lymphoma, and hepatocellular carcinoma (HCC) in different Phase I trials (US National Library of Medicine, 2011; US National Library of Medicine, 2018a; US National Library of Medicine, 2018b). A pilot trial in patients with solid tumors refractory to treatment detected a decrease in the expression of HIF-1α mRNA in four of the six patients (Jeong et al., 2014). In addition, HIF-1α protein expression and HIF-1α target genes VEGF, CA-IX, GLUT-1, and PDK-1 decreased in two of these patients. The decrease in HIF-1α mRNA levels varied between 7%–94%, and the decrease in HIF-1α protein levels varied between 35%–83%. However, there was a surprising increase in HIF-1α mRNA by 111% in one patient and an increase in HIF-1α protein levels by 113% in another patient. Despite that, the study results were evaluated as preliminary proof for the suppression of HIF-1α in the tumor tissues of the patients with the use of EZN-2968.

Wu et al. observed that the synthetic locked nucleic acid form of EZN-2968 (RO7070179) sufficiently decreased the expression of HIF-1α and VEGF at the protein level and induced an apoptotic response when administered at a dose of 10 mg/kg in an orthotopic hepatocellular cancer (HCC) mouse model. This dose led to a 76% decrease in the expression of HIF-1α mRNA in the tumor samples, and increased apoptosis. When they administered RO7070179 to the same model at a dose of 3 mg/kg, HIF-1α mRNA declined by 53%, but a decrease at the protein level could not be achieved, nor the induction of apoptosis. They evaluated these results as the necessity of reducing HIF-1α mRNA level by at least 75% to achieve a clinically relevant response. The phase 1b study of the same group could not meet the primary endpoint of reducing HIF-1α mRNA with one cycle of RO7070179 administration in HCC patients. However, one of the patients responded very well starting from the first cycle of the treatment and maintained the responsiveness till the 12th cycle of the therapy. Even this patient’s first cycle of therapy achieved a decline in HIF-1α mRNA levels by 10%–81%, depending on the biopsy site. Although the progress of this super-responder patient suggested that the synthetic locked nucleic acid form of EZN-2968 may be effective in HCC, further studies in large patient groups are needed for validation (Wu et al., 2019). Nonetheless, the study also pointed out the importance of exploring intra-tumoral heterogeneity in the expression of HIF-1α.

Apart from EZN-2968, almost all the drugs being tested in clinical trials as HIF-1α inhibitors are already approved anticancer agents which indirectly inhibit HIF-1α in addition to their primary mechanism of anticancer action. Among these agents, camptothecins downregulate HIF-1α *via* HIF-1α-targeting miRNAs (Bertozzi et al., 2014), and rapamycin analogs suppress the expression of HIF-1α *via* inhibition of mTOR (Masoud and Li, 2015; Muz et al., 2015). Due to these actions, camptothecins (irinotecan and topotecan) and rapamycin analogs (everolimus and temsirolimus) started to be tested as a part of combination chemotherapy regimens, especially where an antiangiogenic agent like bevacizumab, aflibercept or sorafenib is involved (US National Library of Medicine, 2012; US National Library of Medicine, 2014; US National Library of Medicine, 2017a; US National Library of Medicine, 2017b). This strategy may prevent the induction of HIF-1α by antiangiogenic agents and potentiate the anti-tumor action. A phase one trial aimed to investigate whether chronic use of oral topotecan as a single agent suppresses the expression of HIF-1α and angiogenesis in metastatic tumors with the overexpression of HIF-1α (US National Library of Medicine, 2017c). Moreover, the combination of rapamycin and irinotecan has also been tested for synergism in anti-HIF-1α action and antiangiogenic effect in refractory solid tumors (US National Library of Medicine, 2019). However, the results are not posted also for these trials yet. It should also be noted that a trial that tested the combination of everolimus with multikinase inhibitor sorafenib in advanced solid tumors was suspended due to toxicity (US National Library of Medicine, 2017b).

In addition to camptothecins and rapamycin analogs, digital glucoside digoxin and estradiol metabolite 2-methoxyestradiol (2ME2) have been tested in a multitude of clinical trials, since they suppress the translation of HIF-1α. Digoxin showed promising results in androgen-dependent prostate cancer (US National Library of Medicine, 2016). Digoxin was also tested for its pharmacodynamic effects in newly diagnosed operable breast cancer in a phase two trial (Bardia et al., 2013; US National Library of Medicine, 2020a). This trial was one of the few that explicitly stated the level of HIF-1α suppression aimed with a HIF-1 inhibitor. The trial investigated the expression of HIF-1α and HIF-1α regulated genes CA-9, VEGF, and GLUT by immunohistochemistry and mRNA-sequencing in the tumor tissue of breast cancer patients who received daily digoxin for 14 days or no therapy preoperatively. A 33% reduction in HIF-1α expression was regarded as a clinically admissible response. However, the study results are not published yet. Two recent trials are recruiting patients to evaluate digoxin in other solid tumors (US National Library of Medicine, 2021a; US National Library of Medicine, 2022a). On the other hand, 2ME2 exhibited poor tolerability and efficacy in phase two clinical trials (Harrison et al., 2011; Bruce et al., 2012).

Destabilization of HIF-1α is another mechanism by which indirectly acting agents inhibit HIF-1α. Geldanamycin and its derivatives destabilize HIF-1α *via* inhibition of heat shock protein 90, which is vital for the stability of HIF-1α. Although the efficacy of these drugs has not been proved in clinical trials yet, they seem to have potential in cancer therapy (Bisht et al., 2003; Liu et al., 2015; Weber et al., 2017). The acetylation status of the HIF-1α/p300 complex is also important for the stability of HIF-1α. Histone deacetylase (HDAC) inhibitors destabilize HIF-1α (Fath et al., 2006). Therefore, HDAC inhibitor romidepsin has been tested in several cancers for this action (US National Library of Medicine, 2022b; US National Library of Medicine, 2022c; US National Library of Medicine, 2023).

Anthracyclines, widely used chemotherapeutics in cancer, inhibit HIF-1α action by impairing the binding of HIF-1α to DNA (Lee et al., 2009). The peptide antibiotic echinomycin inhibits HIF-1α with a similar mechanism. Although echinomycin exhibited disappointing results in clinical trials, its nano-liposomal formulations are expected to show a better pharmacokinetic profile and efficacy (Bailey et al., 2020). Some other drugs, like proteasome inhibitor bortezomib, inhibit HIF-1α at multiple levels. Bortezomib leads to the accumulation of ubiquitinated HIF-1α, devoid of the binding ability to p300 and induction of target genes (Shin et al., 2008). Additionally, bortezomib suppresses the expression of HIF-1α *via* inhibition of PI3K/Akt/mTOR and MAPK pathways (Befani et al., 2012). These actions make it an attractive anticancer agent, and the number of clinical trials that test bortezomib’s anticancer efficacy is increasing.

In gastric cancer, camptothecins such as irinotecan and anthracyclines like epirubicin are already used as a part of combination therapy in the clinic. However, their main anticancer action is not through HIF-1α inhibition but topoisomerase inhibition (Wagner et al., 2017). In addition to these, the efficacy of mTOR inhibitors in gastric cancer has been tested in several clinical trials (US National Library of Medicine, 2013a; US National Library of Medicine, 2016b; US National Library of Medicine, 2020b) (Table 2). In one of these clinical trials, mTOR inhibitor everolimus (RAD001) was evaluated to determine the tolerable dose in combination with XELOX (Capecitabine and Oxaliplatin) with an emphasis on its HIF-1α inhibitory action (US National Library of Medicine, 2020c). Although the tolerable doses were determined, the regimen’s efficacy should be evaluated with new trials.

**TABLE 2:** Selected clinical trials registered to ClinicalTrials.gov testing HIF-1α inhibitors in gastric cancer.

Start-end date	Drugs	Condition	Phase	Status	Trial id
Rapamycin analogs (suppress the expression of HIF-1α *via* inhibition of mTOR)					
2007–2009	Everolimus	Advanced gastric cancer	II	Compl.	NCT00519324
2008–2012	Everolimus in combination with Cisplatin, 5-FU, Leucovorin	Metastatic gastric cancer	II	Compl.	NCT00632268
2009–2014	Everolimus	Previously treated unresectable or metastatic esophageal cancer or stomach cancer.	II	Compl.	NCT00985192
2010–2013	Everolimus in combination with XELOX	Advanced gastric cancer	I	Compl.	NCT01049620
Histone Deacetylase (HDAC) inhibitors (destabilize HIF-1α)					
2006–2013	Vorinostat, in combination with Irinotecan, Fluorouracil, and Leucovorin	Advanced upper gastrointestinal cancers	I	Compl.	NCT00537121
2009–2013	Oral HDAC Inhibitor LBH589	Metastatic gastric cancer	II	Termin.	NCT01528501
Proteasome Inhibitors (suppress HIF-1α at multiple levels)					
2003–2013	Bortezomib with or without Irinotecan	Cancer of the gastroesophageal junction or stomach	II	Compl.	NCT00061932
2005–2005	Bortezomib, with Fluorouracil, and Leucovorin	Metastatic or unresectable stomach cancer	II	Termin.	NCT00103103

Compl.: completed, Recruit.: recruiting, Termin.: terminated.

Moreover, HDAC inhibitors and proteasome inhibitors have been evaluated in several clinical trials for treating gastric cancer without emphasizing HIF-1α inhibitory action. One trial with HDAC inhibitor LBH589 was terminated (US National Library of Medicine, 2013b), and the results for another trial with HDAC inhibitor vorinostat have not been posted (US National Library of Medicine, 2013c). Bortezomib has been evaluated as a single agent and combined with 5-FU plus leucovorin or irinotecan in gastric cancer. One trial which tested bortezomib in combination with 5-FU plus leucovorin was terminated (US National Library of Medicine, 2010). Another trial reported the ineffectiveness of bortezomib alone or in combination with irinotecan (Ocean et al., 2014; US National Library of Medicine, 2021b).

## 9 Challenges in targeting HIF-1α and future directions

Incorporating molecularly targeted agents improved the success rate substantially in treating cancer patients with target positivity. However, overcoming chemoresistance is also an obstacle for molecular targeted agents, like conventional chemotherapeutics (Mitani and Kawakami, 2020; Baxter et al., 2021). Excluding the common mechanisms for multidrug resistance, redundancy in the signaling pathways is an important resistance mechanism to molecularly targeted agents in cancer cells (Holohan et al., 2013). Regarding the fact that other HIF-α isoforms, especially HIF-2α, may act as redundant mediators to HIF-1α (Koh et al., 2011), the development of resistance to HIF-1α inhibitors *via* redundancy emerges as a future challenge. The redundancy in HIF signaling may also explain the failure of many HIF-1α inhibitors tested in clinical trials. Currently, several HIF-2α inhibitors are also being tested in clinical trials (Choi et al., 2021), and HIF-2α inhibitor belzutifan was approved for use in VHL-associated cancers (Sheridan, 2021). Hence, the combination of HIF-1α inhibitors with HIF-2α inhibitors or the development of dual HIF-1α/HIF-2α inhibitors may be devised as a strategy to surpass the redundancy in HIF signaling.

The second challenge may be to suppress HIF-1α to a level sufficient to block HIF-1α target pathways and induce an anticancer action. As mentioned earlier, a study in HCC xenograft mouse models suggested that at least a 75% decrement in HIF-1α mRNA would be required to achieve a significant decline in the HIF-1α protein levels and to induce an apoptotic effect (Wu et al., 2019). Achieving a similar level of decrement in HIF-1α expression would also be one of the primary endpoints for patients undergoing treatment with HIF-1α inhibitors. Unfortunately, few clinical studies reported the extent of the decline in the tumor HIF-1α expression after using HIF-1α inhibitors. Detailed reporting of the study results, and analysis of the correlation between percent inhibition in HIF-1α expression and clinical response, are of critical value to determine primary endpoint for HIF-1α suppression in a reliable manner. Nonetheless, the study by Jeong et al. suggested that the efficacy of EZN-2968 to suppress HIF-1α mRNA may exhibit a high interpatient variability, ranging between 7%–94% (Jeong et al., 2014). The reasons behind this high variability in efficacy also worth further exploration.

P53 mutation status emerges as one of the factors with a high potential to determine the efficacy of the HIF-1α inhibitors. Rohwer et al. demonstrated that a functional p53 is required to revert chemoresistance to 5-FU by silencing HIF-1α with siRNAs in gastric cancer cell lines (Rohwer et al., 2010). These observations suggest that patients with p53 mutant tumors may not respond to HIF-1α inhibitors. This possibility should be addressed in future trials.

Intra-tumoral heterogeneity in hypoxia and a non-homogenous distribution of drugs within the tumor mass may pose further limitations to the effect of HIF-1α inhibitors. Hypoxia occurs mainly in the tumor territories distant from the blood vessels (Martin et al., 2016). These territories are also distant from the reach of systemic drugs. Therefore, the distribution of the HIF-1α inhibitors to the most hypoxic niches would be limited due to physical constraints. Moreover, the acidic microenvironment in the hypoxic niches may cause the entrapment of HIF-1α inhibitors in the extracellular space, like chemotherapeutics, limiting their intracellular actions (Jing et al., 2019). Hence, the intra-tumoral heterogeneity in HIF-1α expression and the distribution of HIF-1α inhibitors should be considered as determinators of therapeutic response.

Advancements in single-cell and spatially resolved genomic technologies enabled an in-depth investigation of intra-tumoral heterogeneity in cellular and molecular processes (Hunter et al., 2021; Longo et al., 2021; Wu et al., 2021). Therefore, single-cell genomics and spatial transcriptomics analysis in tumor specimens would allow a thorough exploration of intra-tumoral heterogeneity in HIF-1α and target genes. Moreover, this strategy can identify new predictive markers for response to HIF-1α inhibitors and reveal new therapeutic targets to block HIF-1α signaling.

Cellular senescence is the last but not the least limitation for the action of HIF-1α inhibitors in surpassing cancer chemoresistance. It has long been known that conventional chemotherapeutics act primarily on rapidly proliferating cells and senescent cells are less prone to their anti-cancer actions. Despite that, senescent cells were not usually considered as players in chemoresistance since senescence was regarded as a permanent and non-proliferative state previously. However, growing evidence indicates that senescent cancer cells can exit from this state and generate new cells with more aggressive phenotypes and stem-cell characteristics (Mongiardi et al., 2021). Hence, senescence is now mentioned as a new hallmark for metastasis, tumor progression, and chemoresistance in cancer (Hanahan, 2022).

Hypoxia comes forth as a prominent inducer of senescence in cancer cells. Senescent cancer cells are resistant to the apoptotic actions of chemotherapeutics, mainly due to the overexpression of antiapoptotic proteins. Hence, hypoxia-induced senescence in hypoxic niches constitutes a major mechanism of chemoresistance in tumors. Since HIF-1α is involved in hypoxia-induced senescence by activating cyclin-dependent kinase inhibitors p21_CIP1_ and p27_KIP1_ or inhibiting M-phase inducer CDC25A, anti-HIF-1α strategies may increase the action of anti-cancer agents by abating the senescent phenotype. However, HIF-independent processes are also involved in hypoxia-induced senescence (Otero-Albiol and Carnero, 2021). Moreover, both conventional chemotherapeutics and molecular-targeted agents, mainly tyrosine-kinase inhibitors are potent inducers of senescence in cancer (Mongiardi et al., 2021). These factors can limit the efficacy of anti-HIF-1α agents in surpassing chemoresistance. Nonetheless, we are witnessing growing efforts to explore the benefit of senolytics, agents which aim to kill senescent cells, as an adjunct to anti-cancer therapy (Short et al., 2019; Carpenter et al., 2021). Some of the drug groups mentioned as senolytics, like HDAC inhibitors, which also have anti-HIF-1α action may be promising to overcome therapy resistance in cancer. Yet, the sparsity of studies on senescence and use of senolytics in gastric cancer point to a long way ahead to device these strategies in the clinic.

## 10 Conclusion

HIF-1α is a key inducer of stemness and resistance to systemic therapy in gastric cancer. Therefore, agents that directly or indirectly inhibit HIF-1α signaling may become an effective strategy in gastric cancer treatment in the future. However, further efforts are required to unveil the intricate molecular mechanisms by which HIF-1α reprograms gastric cancer cells into stem cells and potentiates chemoresistance. Also, well-designed clinical trials addressing potential challenges with HIF-1α inhibitors are essential. Such efforts may translate efficacious HIF-1α inhibitors into the clinic and uncover new molecular targets in the HIF-1α signaling pathway to increase anticancer efficacy and overcome chemoresistance in gastric cancer treatment.

## References

[B1] Afify S. M. Seno M. (2019). Conversion of stem cells to cancer stem cells: Undercurrent of cancer initiation. Cancers 11 (3), 345. 10.3390/cancers11030345 30862050 PMC6468812

[B2] Amellem O. Pettersen E. O. (1991). Cell inactivation and cell cycle inhibition as induced by extreme hypoxia: The possible role of cell cycle arrest as a protection against hypoxia-induced lethal damage. Cell Prolif. 24 (2), 127–141. 10.1111/j.1365-2184.1991.tb01144.x 2009318

[B3] Ansari S. Gantuya B. Tuan V. P. Yamaoka Y. (2018). Diffuse gastric cancer: A summary of analogous contributing factors for its molecular pathogenicity. Int. J. Mol. Sci. 19 (8), 2424. 10.3390/ijms19082424 30115886 PMC6121269

[B4] Ayob A. Z. Ramasamy T. S. (2018). Cancer stem cells as key drivers of tumour progression. J. Biomed. Sci. 25 (1), 20. 10.1186/s12929-018-0426-4 29506506 PMC5838954

[B5] Bailey C. M. Liu Y. Peng G. Zhang H. He M. Sun D. (2020). Liposomal formulation of HIF-1α inhibitor echinomycin eliminates established metastases of triple-negative breast cancer. Nanomedicine 29, 102278. 10.1016/j.nano.2020.102278 32738299 PMC7508926

[B6] Bardia A. Santa-Maria C. A. Jacobs L. K. Cimino-Mathews A. Huang P. Russell S. (2013). Digoxin as an inhibitor of global hypoxia inducible factor-1α (HIF1α) expression and downstream targets in breast cancer: Dig-HIF1 pharmacodynamic trial. J. Clin. Oncol. 31 (15), TPS1144.

[B7] Barnhart B. C. Simon M. C. (2007). Metastasis and stem cell pathways. Cancer Metastasis Rev. 26 (2), 261–271. 10.1007/s10555-007-9053-3 17647111 PMC3215288

[B8] Baxter M. A. Middleton F. Cagney H. P. Petty R. D. (2021). Resistance to immune checkpoint inhibitors in advanced gastro-oesophageal cancers. Br. J. Cancer 125 (8), 1068–1079. 10.1038/s41416-021-01425-7 34230609 PMC8505606

[B9] Becerril-Rico J. Alvarado-Ortiz E. Toledo-Guzmán M. E. Pelayo R. Ortiz-Sánchez E. (2021). The cross talk between gastric cancer stem cells and the immune microenvironment: A tumor-promoting factor. Stem Cell Res. Ther. 12 (1), 498. 10.1186/s13287-021-02562-9 34503571 PMC8428093

[B10] Befani C. D. Vlachostergios P. J. Hatzidaki E. Patrikidou A. Bonanou S. Simos G. (2012). Bortezomib represses HIF-1α protein expression and nuclear accumulation by inhibiting both PI3K/Akt/TOR and MAPK pathways in prostate cancer cells. J. Mol. Med. Berl. 90 (1), 45–54. 10.1007/s00109-011-0805-8 21909688

[B11] Bekaii-Saab T. El-Rayes B. (2017). Identifying and targeting cancer stem cells in the treatment of gastric cancer. Cancer 123 (8), 1303–1312. 10.1002/cncr.30538 28117883 PMC5412889

[B12] Berlth F. Bollschweiler E. Drebber U. Hoelscher A. H. Moenig S. (2014). Pathohistological classification systems in gastric cancer: Diagnostic relevance and prognostic value. World J. Gastroenterol. 20 (19), 5679–5684. 10.3748/wjg.v20.i19.5679 24914328 PMC4024777

[B13] Bertozzi D. Marinello J. Manzo S. G. Fornari F. Gramantieri L. Capranico G. (2014). The natural inhibitor of DNA topoisomerase I, camptothecin, modulates HIF-1α activity by changing miR expression patterns in human cancer cells. Mol. Cancer Ther. 13 (1), 239–248. 10.1158/1535-7163.MCT-13-0729 24252850

[B14] Bisht K. S. Bradbury C. M. Mattson D. Kaushal A. Sowers A. Markovina S. (2003). Geldanamycin and 17-Allylamino-17-demethoxygeldanamycin potentiate the *in vitro* and *in vivo* radiation response of cervical tumor cells via the heat shock protein 90-mediated intracellular signaling and cytotoxicity. Cancer Res. 63 (24), 8984–8995. 14695217

[B15] Branco-Price C. Zhang N. Schnelle M. Evans C. Katschinski D. M. Liao D. (2012). Endothelial cell HIF-1α and HIF-2α differentially regulate metastatic success. Cancer Cell 21 (1), 52–65. 10.1016/j.ccr.2011.11.017 22264788 PMC3334270

[B16] Bruce J. Y. Eickhoff J. Pili R. Logan T. Carducci M. Arnott J. (2012). A phase II study of 2-methoxyestradiol nanocrystal colloidal dispersion alone and in combination with sunitinib malate in patients with metastatic renal cell carcinoma progressing on sunitinib malate. Invest. New Drugs 30 (2), 794–802. 10.1007/s10637-010-9618-9 21174224 PMC3191229

[B17] Bubnovskaya L. Kovelskaya A. Gumenyuk L. Ganusevich I. Mamontova L. Mikhailenko V. (2014). Disseminated tumor cells in bone marrow of gastric cancer patients: Correlation with tumor hypoxia and clinical relevance. J. Oncol. 2014, 582140. 10.1155/2014/582140 24669218 PMC3942335

[B18] Bubnovskaya L. Osinsky D. Trachevsky V. Naleskina L. Kovelskaya A. Gumenyuk L. (2014). Premorphological alterations in gastric mucosa in patients with gastric cancer: Hypoxia level assessed by 31P NMR spectroscopy. Exp. Oncol. 36 (4), 271–275. 25537223

[B19] Bubnovskaya L. Osinsky D. (2020). Tumor microenvironment and metabolic factors: Contribution to gastric cancer. Exp. Oncol. 42 (1), 2–10. 10.32471/exp-oncology.2312-8852.vol-42-no-1.14056 32231198

[B20] Burroughs S. K. Kaluz S. Wang D. Wang K. Van Meir E. G. Wang B. (2013). Hypoxia inducible factor pathway inhibitors as anticancer therapeutics. Future Med. Chem. 5 (5), 553–572. 10.4155/fmc.13.17 23573973 PMC3871878

[B21] Carcereri de Prati A. Butturini E. Rigo A. Oppici E. Rossin M. Boriero D. (2017). Metastatic breast cancer cells enter into dormant state and express cancer stem cells phenotype under chronic hypoxia. J. Cell Biochem. 118 (10), 3237–3248. 10.1002/jcb.25972 28262977

[B22] Carnero A. Lleonart M. (2016). The hypoxic microenvironment: A determinant of cancer stem cell evolution. Bioessays 38 (1), S65–S74. 10.1002/bies.201670911 27417124

[B23] Carpenter V. J. Saleh T. Gewirtz D. A. (2021). Senolytics for cancer therapy: Is all that glitters really gold? Cancers (Basel) 13 (4), 723. 10.3390/cancers13040723 33578753 PMC7916462

[B24] Chaplin D. J. Durand R. E. Olive P. L. (1986). Acute hypoxia in tumors: Implications for modifiers of radiation effects. Int. J. Radiat. Oncol. Biol. Phys. 12 (8), 1279–1282. 10.1016/0360-3016(86)90153-7 3759546

[B25] Chen L. Shi Y. Yuan J. Han Y. Qin R. Wu Q. (2014). HIF-1 alpha overexpression correlates with poor overall survival and disease-free survival in gastric cancer patients post-gastrectomy. PLOS ONE 9 (3), e90678. 10.1371/journal.pone.0090678 24614305 PMC3948685

[B26] Choi W. S. W. Boland J. Lin J. (2021). Hypoxia-inducible factor-2α as a novel target in renal cell carcinoma. J. Kidney Cancer VHL 8 (2), 1–7. 10.15586/jkcvhl.v8i1.170 PMC8033537 33868900

[B27] Cisło M. Filip A. A. Arnold Offerhaus G. J. Ciseł B. Rawicz-Pruszyński K. Skierucha M. (2018). Distinct molecular subtypes of gastric cancer: From laurén to molecular pathology. Oncotarget 9 (27), 19427–19442. 10.18632/oncotarget.24827 29721214 PMC5922408

[B28] Correa P. Piazuelo M. B. (2012). The gastric precancerous cascade. J. Dig. Dis. 13 (1), 2–9. 10.1111/j.1751-2980.2011.00550.x 22188910 PMC3404600

[B29] Cui C. P. Wong C. C. Kai A. K. Ho D. W. Lau E. Y. Tsui Y. M. (2017). SENP1 promotes hypoxia-induced cancer stemness by HIF-1α deSUMOylation and SENP1/HIF-1α positive feedback loop. Gut 66 (12), 2149–2159. 10.1136/gutjnl-2016-313264 28258134 PMC5749365

[B30] Danet G. H. Pan Y. Luongo J. L. Bonnet D. A. Simon M. C. (2003). Expansion of human SCID-repopulating cells under hypoxic conditions. J. Clin. Invest. 112 (1), 126–135. 10.1172/JCI17669 12840067 PMC162287

[B31] Das B. Tsuchida R. Malkin D. Koren G. Baruchel S. Yeger H. (2008). Hypoxia enhances tumor stemness by increasing the invasive and tumorigenic side population fraction. Stem Cells 26 (7), 1818–1830. 10.1634/stemcells.2007-0724 18467664

[B32] DeVita V. T. Jr. Chu E. (2008). A history of cancer chemotherapy. Cancer Res. 68 (21), 8643–8653. 10.1158/0008-5472.CAN-07-6611 18974103

[B33] Eales K. L. Hollinshead K. E. Tennant D. A. (2016). Hypoxia and metabolic adaptation of cancer cells. Oncogenesis 5 (1), e190. 10.1038/oncsis.2015.50 26807645 PMC4728679

[B34] Emami Nejad A. Najafgholian S. Rostami A. Sistani A. Shojaeifar S. Esparvarinha M. (2021). The role of hypoxia in the tumor microenvironment and development of cancer stem cell: A novel approach to developing treatment. Cancer Cell Int. 21 (1), 62. 10.1186/s12935-020-01719-5 33472628 PMC7816485

[B35] Fath D. M. Kong X. Liang D. Lin Z. Chou A. Jiang Y. (2006). Histone deacetylase inhibitors repress the transactivation potential of hypoxia-inducible factors independently of direct acetylation of HIF-alpha. J. Biol. Chem. 281 (19), 13612–13619. 10.1074/jbc.M600456200 16543236 PMC1564196

[B36] Fujikuni N. Yamamoto H. Tanabe K. Naito Y. Sakamoto N. Tanaka Y. (2014). Hypoxia-mediated CD24 expression is correlated with gastric cancer aggressiveness by promoting cell migration and invasion. Cancer Sci. 105 (11), 1411–1420. 10.1111/cas.12522 25174257 PMC4462374

[B37] Gerweck L. E. Vijayappa S. Kozin S. (2006). Tumor pH controls the *in vivo* efficacy of weak acid and base chemotherapeutics. Mol. Cancer Ther. 5 (5), 1275–1279. 10.1158/1535-7163.MCT-06-0024 16731760

[B38] Griffiths E. A. Pritchard S. A. Valentine H. R. Whitchelo N. Bishop P. W. Ebert M. P. (2007). Hypoxia-inducible factor-1alpha expression in the gastric carcinogenesis sequence and its prognostic role in gastric and gastro-oesophageal adenocarcinomas. Br. J. Cancer 96 (1), 95–103. 10.1038/sj.bjc.6603524 17179985 PMC2360219

[B39] Gullo I. Carneiro F. Oliveira C. Almeida G. M. (2018). Heterogeneity in gastric cancer: From pure morphology to molecular classifications. Pathobiology 85 (1-2), 50–63. 10.1159/000473881 28618420

[B40] Guo J. Wang B. Fu Z. Wei J. Lu W. (2016). Hypoxic microenvironment induces EMT and upgrades stem-like properties of gastric cancer cells. Technol. Cancer Res. Treat. 15 (1), 60–68. 10.1177/1533034614566413 25601854

[B41] Hanahan D. (2022). Hallmarks of cancer: New dimensions. Cancer Discov. 12 (1), 31–46. 10.1158/2159-8290.CD-21-1059 35022204

[B42] Harrison M. R. Hahn N. M. Pili R. Oh W. K. Hammers H. Sweeney C. (2011). A phase II study of 2-methoxyestradiol (2ME2) NanoCrystal® dispersion (NCD) in patients with taxane-refractory, metastatic castrate-resistant prostate cancer (CRPC). Invest. New Drugs 29 (6), 1465–1474. 10.1007/s10637-010-9455-x 20499131 PMC3042040

[B43] Hashimoto K. Aoyagi K. Isobe T. Kouhuji K. Shirouzu K. (2014). Expression of CD133 in the cytoplasm is associated with cancer progression and poor prognosis in gastric cancer. Gastric Cancer 17 (1), 97–106. 10.1007/s10120-013-0255-9 23558457 PMC3889295

[B44] Heddleston J. M. Li Z. McLendon R. E. Hjelmeland A. B. Rich J. N. (2009). The hypoxic microenvironment maintains glioblastoma stem cells and promotes reprogramming towards a cancer stem cell phenotype. Cell Cycle 8 (20), 3274–3284. 10.4161/cc.8.20.9701 19770585 PMC2825672

[B45] Hill R. P. Marie-Egyptienne D. T. Hedley D. W. (2009). Cancer stem cells, hypoxia and metastasis. Semin. Radiat. Oncol. 19 (2), 106–111. 10.1016/j.semradonc.2008.12.002 19249648

[B46] Holohan C. Van Schaeybroeck S. Longley D. B. Johnston P. G. (2013). Cancer drug resistance: An evolving paradigm. Nat. Rev. Cancer 13 (10), 714–726. 10.1038/nrc3599 24060863

[B47] Huang L. Wu R. L. Xu A. M. (2015). Epithelial-mesenchymal transition in gastric cancer. Am. J. Transl. Res. 7 (11), 2141–2158. 26807164 PMC4697696

[B48] Hui E. P. Chan A. T. Pezzella F. Turley H. To K. F. Poon T. C. (2002). Coexpression of hypoxia-inducible factors 1alpha and 2alpha, carbonic anhydrase IX, and vascular endothelial growth factor in nasopharyngeal carcinoma and relationship to survival. Clin. Cancer Res. 8 (8), 2595–2604. 12171890

[B49] Hunter M. V. Moncada R. Weiss J. M. Yanai I. White R. M. (2021). Spatially resolved transcriptomics reveals the architecture of the tumor-microenvironment interface. Nat. Commun. 12 (1), 6278. 10.1038/s41467-021-26614-z 34725363 PMC8560802

[B50] Jeong W. Rapisarda A. Park S. R. Kinders R. J. Chen A. Melillo G. (2014). Pilot trial of EZN-2968, an antisense oligonucleotide inhibitor of hypoxia-inducible factor-1 alpha (HIF-1α), in patients with refractory solid tumors. Cancer Chemother. Pharmacol. 73 (2), 343–348. 10.1007/s00280-013-2362-z 24292632 PMC8375568

[B51] Jing X. Yang F. Shao C. Wei K. Xie M. Shen H. (2019). Role of hypoxia in cancer therapy by regulating the tumor microenvironment. Mol. Cancer 18 (1), 157. 10.1186/s12943-019-1089-9 31711497 PMC6844052

[B52] Joshi S. S. Badgwell B. D. (2021). Current treatment and recent progress in gastric cancer. CA Cancer J. Clin. 71 (3), 264–279. 10.3322/caac.21657 33592120 PMC9927927

[B53] Jung J-H. Im S. Jung E. S. Kang C. S. (2013). Clinicopathological implications of the expression of hypoxia-related proteins in gastric cancer. Int. J. Med. Sci. 10 (9), 1217–1223. 10.7150/ijms.6054 23935399 PMC3739021

[B54] Kaelin W. G. Jr. Ratcliffe P. J. (2008). Oxygen sensing by metazoans: The central role of the HIF hydroxylase pathway. Mol. Cell 30 (4), 393–402. 10.1016/j.molcel.2008.04.009 18498744

[B55] Keith B. Simon M. C. (2007). Hypoxia-inducible factors, stem cells, and cancer. Cell 129 (3), 465–472. 10.1016/j.cell.2007.04.019 17482542 PMC3150586

[B56] Kim H. Lin Q. Glazer P. M. Yun Z. (2018). The hypoxic tumor microenvironment *in vivo* selects the cancer stem cell fate of breast cancer cells. Breast Cancer Res. 20 (1), 16. 10.1186/s13058-018-0944-8 29510720 PMC5840770

[B57] Kim J. I. Choi K. U. Lee I. S. Choi Y. J. Kim W. T. Shin D. H. (2015). Expression of hypoxic markers and their prognostic significance in soft tissue sarcoma. Oncol. Lett. 9 (4), 1699–1706. 10.3892/ol.2015.2914 25789026 PMC4356356

[B58] Kitajima Y. Miyazaki K. (2013). The critical impact of HIF-1a on gastric cancer Biology. Cancers (Basel) 5 (1), 15–26. 10.3390/cancers5010015 24216696 PMC3730315

[B59] Koh M. Y. Lemos R. Jr Liu X. Powis G. (2011). The hypoxia-associated factor switches cells from HIF-1α-to HIF-2α-Dependent signaling promoting stem cell characteristics, aggressive tumor growth and invasion. Cancer Res. 71 (11), 4015–4027. 10.1158/0008-5472.CAN-10-4142 21512133 PMC3268651

[B60] Koi M. Boland C. R. (2011). Tumor hypoxia and genetic alterations in sporadic cancers. J. Obstet. Gynaecol. Res. 37 (2), 85–98. 10.1111/j.1447-0756.2010.01377.x 21272156 PMC3079283

[B61] Krstić M. Katić V. (2008). Histological, mucinohistochemical and immunohistochemical features of gastric signet ring cell carcinoma. Vojnosanit. Pregl. 65 (11), 835–838. 10.2298/vsp0811835k 19069715

[B62] Kunz M. Ibrahim S. M. (2003). Molecular responses to hypoxia in tumor cells. Mol. Cancer 2, 23. 10.1186/1476-4598-2-23 12740039 PMC155638

[B63] Lan J. Lu H. Samanta D. Salman S. Lu Y. Semenza G. L. (2018). Hypoxia-inducible factor 1-dependent expression of adenosine receptor 2B promotes breast cancer stem cell enrichment. Proc. Natl. Acad. Sci. U. S. A. 115 (41), E9640–E9648. 10.1073/pnas.1809695115 30242135 PMC6187157

[B64] Lee J. Cristescu R. Kim K. M. Kim K. Kim S. T. Park S. H. (2017). Development of mesenchymal subtype gene signature for clinical application in gastric cancer. Oncotarget 8 (39), 66305–66315. 10.18632/oncotarget.19985 29029513 PMC5630413

[B65] Lee K. Qian D. Z. Rey S. Wei H. Liu J. O. Semenza G. L. (2009). Anthracycline chemotherapy inhibits HIF-1 transcriptional activity and tumor-induced mobilization of circulating angiogenic cells. Proc. Natl. Acad. Sci. U. S. A. 106 (7), 2353–2358. 10.1073/pnas.0812801106 19168635 PMC2650160

[B66] Li K. Dan Z. Nie Y. Q. (2014). Gastric cancer stem cells in gastric carcinogenesis, progression, prevention and treatment. World J. Gastroenterol. 20 (18), 5420–5426. 10.3748/wjg.v20.i18.5420 24833872 PMC4017057

[B67] Liang G. Li S. Du W. Ke Q. Cai J. Yang J. (2017). Hypoxia regulates CD44 expression via hypoxia-inducible factor-1α in human gastric cancer cells. Oncol. Lett. 13 (2), 967–972. 10.3892/ol.2016.5473 28356986 PMC5351347

[B68] Lin S. Ma R. Zheng X-Y. Yu H. Liang X. Lin H. (2014). Meta-analysis of immunohistochemical expression of hypoxia inducible factor-1α as a prognostic role in gastric cancer. World J. gastroenterology 20 (4), 1107–1113. 10.3748/wjg.v20.i4.1107 PMC3921536 24574785

[B69] Liu H. Lu J. Hua Y. Zhang P. Liang Z. Ruan L. (2015). Targeting heat-shock protein 90 with ganetespib for molecularly targeted therapy of gastric cancer. Cell Death Dis. 6 (1), e1595. 10.1038/cddis.2014.555 25590805 PMC4669753

[B70] Liu L. Ning X. Sun L. Zhang H. Shi Y. Guo C. (2008). Hypoxia-inducible factor-1α contributes to hypoxia-induced chemoresistance in gastric cancer. Cancer Sci. 99 (1), 121–128. 10.1111/j.1349-7006.2007.00643.x 17953712 PMC11158535

[B71] Longo S. K. Guo M. G. Ji A. L. Khavari P. A. (2021). Integrating single-cell and spatial transcriptomics to elucidate intercellular tissue dynamics. Nat. Rev. Genet. 22 (10), 627–644. 10.1038/s41576-021-00370-8 34145435 PMC9888017

[B72] Lordick F. Carneiro F. Cascinu S. Fleitas T. Haustermans K. Piessen G. (2022). Gastric cancer: ESMO clinical practice guideline for diagnosis, treatment and follow-up. Ann. Oncol. 33, 1005–1020. 10.1016/j.annonc.2022.07.004 35914639

[B73] Ma J. Zhang L. Ru G. Q. Zhao Z. S. Xu W. J. (2007). Upregulation of hypoxia inducible factor 1alpha mRNA is associated with elevated vascular endothelial growth factor expression and excessive angiogenesis and predicts a poor prognosis in gastric carcinoma. World J. Gastroenterol. 13 (11), 1680–1686. 10.3748/wjg.v13.i11.1680 17461470 PMC4146946

[B74] Mahoney B. P. Raghunand N. Baggett B. Gillies R. J. (2003). Tumor acidity, ion trapping and chemotherapeutics. I. Acid pH affects the distribution of chemotherapeutic agents *in vitro* . Biochem. Pharmacol. 66 (7), 1207–1218. 10.1016/s0006-2952(03)00467-2 14505800

[B75] Marin J. J. G. Perez-Silva L. Macias R. I. R. Asensio M. Peleteiro-Vigil A. Sanchez-Martin A. (2020). Molecular bases of mechanisms accounting for drug resistance in gastric adenocarcinoma. Cancers 12 (8), 2116. 10.3390/cancers12082116 32751679 PMC7463778

[B76] Martin J. D. Fukumura D. Duda D. G. Boucher Y. Jain R. K. (2016). Corrigendum: Reengineering the tumor microenvironment to alleviate hypoxia and overcome cancer heterogeneity. Cold Spring Harb. Perspect. Med. 6 (12), a031195. 10.1101/cshperspect.a031195 27908927 PMC5131752

[B77] Masoud G. N. Li W. (2015). HIF-1α pathway: Role, regulation and intervention for cancer therapy. Acta Pharm. Sin. B 5 (5), 378–389. 10.1016/j.apsb.2015.05.007 26579469 PMC4629436

[B78] Matsumoto K. Arao T. Tanaka K. Kaneda H. Kudo K. Fujita Y. (2009). mTOR signal and hypoxia-inducible factor-1 alpha regulate CD133 expression in cancer cells. Cancer Res. 69 (18), 7160–7164. 10.1158/0008-5472.CAN-09-1289 19738050

[B79] Matsuoka J. Yashiro M. Doi Y. Fuyuhiro Y. Kato Y. Shinto O. (2013). Hypoxia stimulates the EMT of gastric cancer cells through autocrine TGFβ signaling. PLoS One 8 (5), e62310. 10.1371/journal.pone.0062310 23690936 PMC3656884

[B80] Miao S. Wang S. M. Cheng X. Li Y. F. Zhang Q. S. Li G. (2017). Erythropoietin promoted the proliferation of hepatocellular carcinoma through hypoxia induced translocation of its specific receptor. Cancer Cell Int. 17, 119. 10.1186/s12935-017-0494-7 29238266 PMC5725980

[B81] Miao Z. F. Wang Z. N. Zhao T. T. Xu Y. Y. Gao J. Miao F. (2014). Peritoneal milky spots serve as a hypoxic niche and favor gastric cancer stem/progenitor cell peritoneal dissemination through hypoxia-inducible factor 1α. Stem Cells 32 (12), 3062–3074. 10.1002/stem.1816 25142304 PMC4282537

[B82] Mitani S. Kawakami H. (2020). Emerging targeted therapies for HER2 positive gastric cancer that can overcome trastuzumab resistance. Cancers (Basel) 12 (2), 400. 10.3390/cancers12020400 32050652 PMC7072407

[B83] Mongiardi M. P. Pellegrini M. Pallini R. Levi A. Falchetti M. L. (2021). Cancer response to therapy-induced senescence: A matter of dose and timing. Cancers (Basel) 13 (3), 484. 10.3390/cancers13030484 33513872 PMC7865402

[B84] Morrison S. J. Csete M. Groves A. K. Melega W. Wold B. Anderson D. J. (2000). Culture in reduced levels of oxygen promotes clonogenic sympathoadrenal differentiation by isolated neural crest stem cells. J. Neurosci. 20 (19), 7370–7376. 10.1523/JNEUROSCI.20-19-07370.2000 11007895 PMC6772795

[B85] Muz B. de la Puente P. Azab F. Azab A. K. (2015). The role of hypoxia in cancer progression, angiogenesis, metastasis, and resistance to therapy. Hypoxia (Auckl) 3, 83–92. 10.2147/HP.S93413 27774485 PMC5045092

[B86] Najafi M. Farhood B. Mortezaee K. Kharazinejad E. Majidpoor J. Ahadi R. (2020). Hypoxia in solid tumors: A key promoter of cancer stem cell (CSC) resistance. J. Cancer Res. Clin. Oncol. 146 (1), 19–31. 10.1007/s00432-019-03080-1 31734836 PMC11804417

[B87] Nakamura J. Kitajima Y. Kai K. Mitsuno M. Ide T. Hashiguchi K. (2009). Hypoxia-inducible factor-1alpha expression predicts the response to 5-fluorouracil-based adjuvant chemotherapy in advanced gastric cancer. Oncol. Rep. 22 (4), 693–699. 10.3892/or_00000489 19724845

[B88] Nikolaou M. Pavlopoulou A. Georgakilas A. G. Kyrodimos E. (2018). The challenge of drug resistance in cancer treatment: A current overview. Clin. Exp. Metastasis 35 (4), 309–318. 10.1007/s10585-018-9903-0 29799080

[B89] Ocean A. J. Christos P. Sparano J. A. Shah M. A. Yantiss R. K. Cheng J. (2014). Phase II trial of bortezomib alone or in combination with irinotecan in patients with adenocarcinoma of the gastroesophageal junction or stomach. Invest. New Drugs 32 (3), 542–548. 10.1007/s10637-014-0070-0 24526575 PMC4047141

[B90] Okazaki M. Fushida S. Tsukada T. Kinoshita J. Oyama K. Miyashita T. (2018). The effect of HIF-1α and PKM1 expression on acquisition of chemoresistance. Cancer Manag. Res. 10, 1865–1874. 10.2147/CMAR.S166136 30013393 PMC6037278

[B91] Olive P. L. Vikse C. Trotter M. J. (1992). Measurement of oxygen diffusion distance in tumor cubes using a fluorescent hypoxia probe. Int. J. Radiat. Oncol. Biol. Phys. 22 (3), 397–402. 10.1016/0360-3016(92)90840-e 1735668

[B92] Otero-Albiol D. Carnero A. (2021). Cellular senescence or stemness: Hypoxia flips the coin. J. Exp. Clin. Cancer Res. 40 (1), 243. 10.1186/s13046-021-02035-0 34325734 PMC8323321

[B93] Özcan G. (2020). “Clinical development of HIF-1α inhibitors for cancer therapy,” in Medical diagnosis and treatment methods in basic medical sciences. Editor Keskin S. (Lyon: Livre de Lyon), 25–37.

[B94] Pei J. P. Zhang C. D. Yusupu M. Zhang C. Dai D. Q. (2021). Screening and validation of the hypoxia-related signature of evaluating tumor immune microenvironment and predicting prognosis in gastric cancer. Front. Immunol. 12, 705511. 10.3389/fimmu.2021.705511 34249015 PMC8267919

[B95] Qian J. Rankin E. B. (2019). Hypoxia-induced phenotypes that mediate tumor heterogeneity. Adv. Exp. Med. Biol. 1136, 43–55. 10.1007/978-3-030-12734-3_3 31201715 PMC7039393

[B96] Rao X. Zhang C. Luo H. Zhang J. Zhuang Z. Liang Z. (2022). Targeting gastric cancer stem cells to enhance treatment response. Cells 11 (18), 2828. 10.3390/cells11182828 36139403 PMC9496718

[B97] Ravi R. Mookerjee B. Bhujwalla Z. M. Sutter C. H. Artemov D. Zeng Q. (2000). Regulation of tumor angiogenesis by p53-induced degradation of hypoxia-inducible factor 1α. Genes Dev. 14 (1), 34–44. 10.1101/gad.14.1.34 10640274 PMC316350

[B98] Raz S. Sheban D. Gonen N. Stark M. Berman B. Assaraf Y. G. (2014). Severe hypoxia induces complete antifolate resistance in carcinoma cells due to cell cycle arrest. Cell Death Dis. 5 (2), e1067–e. 10.1038/cddis.2014.39 24556682 PMC3944254

[B99] Riquelme I. Saavedra K. Espinoza J. A. Weber H. García P. Nervi B. (2015). Molecular classification of gastric cancer: Towards a pathway-driven targeted therapy. Oncotarget 6 (28), 24750–24779. 10.18632/oncotarget.4990 26267324 PMC4694793

[B100] Rohwer N. Dame C. Haugstetter A. Wiedenmann B. Detjen K. Schmitt C. A. (2010). Hypoxia-inducible factor 1alpha determines gastric cancer chemosensitivity via modulation of p53 and NF-kappaB. PLOS ONE 5 (8), e12038. 10.1371/journal.pone.0012038 20706634 PMC2919384

[B101] Ruan K. Song G. Ouyang G. (2009). Role of hypoxia in the hallmarks of human cancer. J. Cell Biochem. 107 (6), 1053–1062. 10.1002/jcb.22214 19479945

[B102] Rybinski B. Yun K. (2016). Addressing intra-tumoral heterogeneity and therapy resistance. Oncotarget 7 (44), 72322–72342. 10.18632/oncotarget.11875 27608848 PMC5342165

[B103] Semenza G. L. (2012). Hypoxia-inducible factors in physiology and medicine. Cell 148 (3), 399–408. 10.1016/j.cell.2012.01.021 22304911 PMC3437543

[B104] Senthebane D. A. Rowe A. Thomford N. E. Shipanga H. Munro D. Mazeedi M. (2017). The role of tumor microenvironment in chemoresistance: To survive, keep your enemies closer. Int. J. Mol. Sci. 18 (7), 1586. 10.3390/ijms18071586 28754000 PMC5536073

[B105] Seo E. J. Kim D. K. Jang I. H. Choi E. J. Shin S. H. Lee S. I. (2016). Hypoxia-NOTCH1-SOX2 signaling is important for maintaining cancer stem cells in ovarian cancer. Oncotarget 7 (34), 55624–55638. 10.18632/oncotarget.10954 27489349 PMC5342441

[B106] Sheridan C. (2021). Oncologists greet lumakras: The world's first KRAS inhibitor. Nat. Biotechnol. 39 (9), 1032–1034. 10.1038/s41587-021-01053-9 34504350

[B107] Shin D. H. Chun Y. S. Lee D. S. Huang L. E. Park J. W. (2008). Bortezomib inhibits tumor adaptation to hypoxia by stimulating the FIH-mediated repression of hypoxia-inducible factor-1. Blood 111 (6), 3131–3136. 10.1182/blood-2007-11-120576 18174379

[B108] Short S. Fielder E. Miwa S. von Zglinicki T. (2019). Senolytics and senostatics as adjuvant tumour therapy. EBioMedicine 41, 683–692. 10.1016/j.ebiom.2019.01.056 30737084 PMC6441870

[B109] Singh A. Settleman J. (2010). EMT, cancer stem cells and drug resistance: An emerging axis of evil in the war on cancer. Oncogene 29 (34), 4741–4751. 10.1038/onc.2010.215 20531305 PMC3176718

[B110] Sung H. Ferlay J. Siegel R. L. Laversanne M. Soerjomataram I. Jemal A. (2021). Global cancer statistics 2020: GLOBOCAN estimates of incidence and mortality worldwide for 36 cancers in 185 countries. CA A Cancer J. Clin. 71 (3), 209–249. 10.3322/caac.21660 33538338

[B111] Susman S. Barnoud R. Bibeau F. Borrini F. Pocard M. Tomuleasa C. (2015). The lauren classification highlights the role of epithelial-to-mesenchymal transition in gastric carcinogenesis: An immunohistochemistry study of the STAT3 and adhesion molecules expression. J. Gastrointestin Liver Dis. 24 (1), 77–83. 10.15403/jgld.2014.1121.sus 25822437

[B112] Takaishi S. Okumura T. Tu S. Wang S. S. W. Shibata W. Vigneshwaran R. (2009). Identification of gastric cancer stem cells using the cell surface marker CD44. Stem cells Dayt. Ohio) 27 (5), 1006–1020. 10.1002/stem.30 PMC2746367 19415765

[B113] Tannock I. F. Rotin D. (1989). Acid pH in tumors and its potential for therapeutic exploitation. Cancer Res. 49 (16), 4373–4384. 2545340

[B114] Tong W-W. Tong G-H. Liu Y. (2018). Cancer stem cells and hypoxia-inducible factors (Review). Int. J. Oncol. 53 (2), 469–476. 10.3892/ijo.2018.4417 29845228

[B115] Traverso N. Ricciarelli R. Nitti M. Marengo B. Furfaro A. L. Pronzato M. A. (2013). Role of glutathione in cancer progression and chemoresistance. Oxid. Med. Cell Longev. 2013, 972913. 10.1155/2013/972913 23766865 PMC3673338

[B116] Trédan O. Galmarini C. M. Patel K. Tannock I. F. (2007). Drug resistance and the solid tumor microenvironment. J. Natl. Cancer Inst. 99 (19), 1441–1454. 10.1093/jnci/djm135 17895480

[B117] Ucaryilmaz Metin C. Ozcan G. (2022). The HIF-1α as a potent inducer of the hallmarks in gastric cancer. Cancers (Basel) 14 (11), 2711. 10.3390/cancers14112711 35681691 PMC9179860

[B118] US National Library of Medicine (2007). Available at: https://clinicaltrials.gov/ct2/home . 10.1080/15360280801989377 28792816

[B119] US National Library of Medicine (2010). ClinicalTrials.gov. Trial ID: NCT00103103 Available at: https://clinicaltrials.gov/ct2/show/NCT00103103 . 10.1080/15360280801989377 28792816

[B120] US National Library of Medicine (2011). ClinicalTrials.gov. Trial ID: NCT00466583 Available at: https://clinicaltrials.gov/ct2/show/NCT00466583 . 10.1080/15360280801989377 28792816

[B121] US National Library of Medicine (2012). ClinicalTrials.gov. Trial ID: NCT00381797 [Available from: https://clinicaltrials.gov/ct2/show/NCT00381797 . 10.1080/15360280801989377 28792816

[B122] US National Library of Medicine (2013a). ClinicalTrials.gov. Trial ID: NCT00632268 Available at: https://clinicaltrials.gov/ct2/show/NCT00632268 . 10.1080/15360280801989377 28792816

[B123] US National Library of Medicine (2013b). ClinicalTrials.gov. Trial ID: NCT01528501 Available at: https://clinicaltrials.gov/ct2/show/NCT01528501 . 10.1080/15360280801989377 28792816

[B124] US National Library of Medicine (2013c). ClinicalTrials.gov. Trial ID: NCT00537121 Available at: https://clinicaltrials.gov/ct2/show/NCT00537121 . 10.1080/15360280801989377 28792816

[B125] US National Library of Medicine (2014). ClinicalTrials.gov. Trial ID: NCT00548418 Available at: https://clinicaltrials.gov/ct2/show/NCT00548418 . 10.1080/15360280801989377 28792816

[B126] US National Library of Medicine (2016b). ClinicalTrials.gov. Trial ID: NCT00519324 Available at: https://clinicaltrials.gov/ct2/show/NCT00519324 . 10.1080/15360280801989377 28792816

[B127] US National Library of Medicine (2016). ClinicalTrials.gov. Trial ID: NCT01162135 Available at: https://clinicaltrials.gov/ct2/show/NCT01162135 . 10.1080/15360280801989377 28792816

[B128] US National Library of Medicine (2017a). ClinicalTrials.gov. Trial ID: NCT01047293 Available at: https://clinicaltrials.gov/ct2/show/NCT01047293 . 10.1080/15360280801989377 28792816

[B129] US National Library of Medicine (2017b). ClinicalTrials.gov. Trial ID: NCT01226056 Available at: https://clinicaltrials.gov/ct2/show/NCT01226056 . 10.1080/15360280801989377 28792816

[B130] US National Library of Medicine (2017c). ClinicalTrials.gov. Trial ID: NCT00117013 Available at: https://clinicaltrials.gov/ct2/show/NCT00117013 . 10.1080/15360280801989377 28792816

[B131] US National Library of Medicine (2018a). ClinicalTrials.gov. Trial ID: NCT01120288 Available at: https://clinicaltrials.gov/ct2/show/NCT01120288 . 10.1080/15360280801989377 28792816

[B132] US National Library of Medicine (2018b). ClinicalTrials.gov. Trial ID: NCT02564614 Available at: https://clinicaltrials.gov/ct2/show/NCT02564614 . 10.1080/15360280801989377 28792816

[B133] US National Library of Medicine (2019). ClinicalTrials.gov. Trial ID: NCT01282697 Available at: https://clinicaltrials.gov/ct2/show/NCT01282697 . 10.1080/15360280801989377 28792816

[B134] US National Library of Medicine (2020a). ClinicalTrials.gov. Trial ID: NCT01763931 Available at: https://clinicaltrials.gov/ct2/show/NCT01763931 . 10.1080/15360280801989377 28792816

[B135] US National Library of Medicine (2020b). ClinicalTrials.gov. Trial ID: NCT00985192 Available at: https://clinicaltrials.gov/ct2/show/NCT00985192 . 10.1080/15360280801989377 28792816

[B136] US National Library of Medicine (2020c). ClinicalTrials.gov. Trial ID: NCT01049620 Available at: https://clinicaltrials.gov/ct2/show/NCT01049620 . 10.1080/15360280801989377 28792816

[B137] US National Library of Medicine (2021a). ClinicalTrials.gov. Trial ID: NCT03889795 Available at: https://clinicaltrials.gov/ct2/show/NCT03889795 . 10.1080/15360280801989377 28792816

[B138] US National Library of Medicine (2021b). ClinicalTrials.gov. Trial ID: NCT00061932 Available at: https://clinicaltrials.gov/ct2/show/NCT00061932 . 10.1080/15360280801989377 28792816

[B139] US National Library of Medicine (2022a). ClinicalTrials.gov. Trial ID: NCT04141995 Available at: https://clinicaltrials.gov/ct2/show/NCT04141995 . 10.1080/15360280801989377 28792816

[B140] US National Library of Medicine (2022b). ClinicalTrials.gov. Trial ID: NCT02512172 Available at: https://clinicaltrials.gov/ct2/show/NCT02512172 . 10.1080/15360280801989377 28792816

[B141] US National Library of Medicine (2022c). ClinicalTrials.gov. Trial ID: NCT01947140 Available at: https://clinicaltrials.gov/ct2/show/NCT01947140 . 10.1080/15360280801989377 28792816

[B142] US National Library of Medicine (2023). ClinicalTrials.gov. Trial ID: NCT01638533 Available at: https://clinicaltrials.gov/ct2/show/NCT01638533 . 10.1080/15360280801989377 28792816

[B143] Valencia-Cervantes J. Huerta-Yepez S. Aquino-Jarquín G. Rodríguez-Enríquez S. Martínez-Fong D. Arias-Montaño J. A. (2019). Hypoxia increases chemoresistance in human medulloblastoma DAOY cells via hypoxia-inducible factor 1α-mediated downregulation of the CYP2B6, CYP3A4 and CYP3A5 enzymes and inhibition of cell proliferation. Oncol. Rep. 41 (1), 178–190. 10.3892/or.2018.6790 30320358 PMC6278548

[B144] Vasan N. Baselga J. Hyman D. M. (2019). A view on drug resistance in cancer. Nature 575 (7782), 299–309. 10.1038/s41586-019-1730-1 31723286 PMC8008476

[B145] Vaupel P. (2008). Hypoxia and aggressive tumor phenotype: Implications for therapy and prognosis. Oncologist 13 (3), 21–26. 10.1634/theoncologist.13-S3-21 18458121

[B146] Wagner A. D. Syn N. L. Moehler M. Grothe W. Yong W. P. Tai B. C. (2017). Chemotherapy for advanced gastric cancer. Cochrane Database Syst. Rev. 8 (8), Cd004064. 10.1002/14651858.CD004064.pub4 28850174 PMC6483552

[B147] Weber H. Valbuena J. R. Barbhuiya M. A. Stein S. Kunkel H. García P. (2017). Small molecule inhibitor screening identifified HSP90 inhibitor 17-AAG as potential therapeutic agent for gallbladder cancer. Oncotarget 8 (16), 26169–26184. 10.18632/oncotarget.15410 28412732 PMC5432248

[B148] Wilson M. M. Weinberg R. A. Lees J. A. Guen V. J. (2020). Emerging mechanisms by which EMT programs control stemness. Trends Cancer 6 (9), 775–780. 10.1016/j.trecan.2020.03.011 32312682

[B149] Wu F. Fan J. He Y. Xiong A. Yu J. Li Y. (2021). Single-cell profiling of tumor heterogeneity and the microenvironment in advanced non-small cell lung cancer. Nat. Commun. 12 (1), 2540. 10.1038/s41467-021-22801-0 33953163 PMC8100173

[B150] Wu J. Contratto M. Shanbhogue K. P. Manji G. A. O'Neil B. H. Noonan A. (2019). Evaluation of a locked nucleic acid form of antisense oligo targeting HIF-1α in advanced hepatocellular carcinoma. World J. Clin. Oncol. 10 (3), 149–160. 10.5306/wjco.v10.i3.149 30949444 PMC6441661

[B151] Yan Y. Zuo X. Wei D. (2015). Concise review: Emerging role of CD44 in cancer stem cells: A promising biomarker and therapeutic target. Stem Cells Transl. Med. 4 (9), 1033–1043. 10.5966/sctm.2015-0048 26136504 PMC4542874

[B152] Yang Y. Meng W. J. Wang Z. Q. (2022). The origin of gastric cancer stem cells and their effects on gastric cancer: Novel therapeutic targets for gastric cancer. Front. Oncol. 12, 960539. 10.3389/fonc.2022.960539 36185219 PMC9520244

[B153] Zhang H. Sun L. Xiao X. Xie R. Liu C. Wang Y. (2014). Krüppel-like factor 8 contributes to hypoxia-induced MDR in gastric cancer cells. Cancer Sci. 105 (9), 1109–1115. 10.1111/cas.12483 25040744 PMC4462403

